# The methyltransferase Suv39h1 links the SUMO pathway to HP1α marking at pericentric heterochromatin

**DOI:** 10.1038/ncomms12224

**Published:** 2016-07-18

**Authors:** Christèle Maison, Delphine Bailly, Jean-Pierre Quivy, Geneviève Almouzni

**Affiliations:** 1 Institut Curie, PSL Research University, CNRS, UMR3664, Equipe Labellisée Ligue contre le Cancer, F-75005 Paris, France; 2 Sorbonne Universités, UPMC Université Paris 06, CNRS, UMR3664, F-75005 Paris, France

## Abstract

The trimethylation of histone H3 on lysine 9 (H3K9me3) – a mark recognized by HP1 that depends on the Suv39h lysine methyltransferases (KMTs) – has provided a basis for the reader/writer model to explain HP1 accumulation at pericentric heterochromatin in mammals. Here, we identify the Suv39h1 paralog, as a unique enhancer of HP1α sumoylation both *in vitro* and *in vivo*. The region responsible for promoting HP1α sumoylation (aa1–167) is distinct from the KMT catalytic domain and mediates binding to Ubc9. Tethering the 1–167 domain of Suv39h1 to pericentric heterochromatin, but not mutants unable to bind Ubc9, accelerates the *de novo* targeting of HP1α to these domains. Our results establish an unexpected feature of Suv39h1, distinct from the KMT activity, with a major role for heterochromatin formation. We discuss how linking Suv39h1 to the SUMO pathway provides conceptual implications for our general view on nuclear domain organization and physiological functions.

## Abstract (web summary)

The Suv39h histone methyltransferases promote trimethylation of histone H3 on lysine 9 (H3K9me3). Here, in the Suv39h1 paralog, the authors identify an enhancer of HP1a sumoylation activity that impacts heterochromatin.

## Graphical abstract

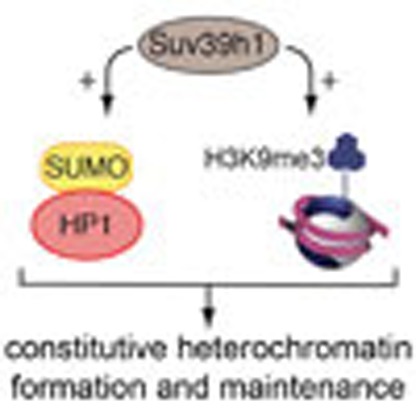

Heterochromatin at pericentric domains is essential for centromere function and genome stability^1^,^2^. Among the major proteins conserved from fission yeast to mammals considered critical in organizing pericentric heterochromatin, the Suv39h lysine methyltransferases (KMTs) and HP1 proteins stand out. Indeed, Suv39h KMTs are responsible for the accumulation at pericentric heterochromatin of histone H3 lysine 9 trimethylation (H3K9me3)^3^,^4^, a mark epigenetically inherited in *S. pombe* (refs 5, 6, 7). In turn, HP1 recognizes and binds H3K9me3 (refs 8, 9). In line with the writer/reader model in the histone code hypothesis^10^,^11^, it contributes to the pericentric enrichment of HP1 and heterochromatin formation. In mice, Suv39h KMTs are encoded in the genome at two loci *Suv39h1* and *Suv39h2* as two paralogs sharing 59% sequence identity^12^. Fluorescence recovery after photobleaching studies^13^,^14^ showed that Suv39h1 is more dynamic at pericentric heterochromatin compared with Suv39h2. Furthermore, they are differentially expressed during early mouse development and in adult tissues^12^,^15^. Thus, while in the current view, Suv39h1 and Suv39h2 are thought to provide essentially redundant activities, these data hint that these paralogs might have distinct properties or a unique function beyond H3K9 methylation. We recently found that in the absence of Suv39h-dependent H3K9me3, HP1α sumoylation could promote its *de novo* targeting to pericentric heterochromatin in mammalian cells^16^. We thus proposed at least two distinct steps in heterochromatin formation: first ‘seeding' requiring HP1 sumoylation, and second amplification and propagation exploiting H3K9me3. The importance in this scheme of a SUMO-dependent step raises the issue of the identification of the SUMO E3 ligase specific for HP1α at pericentric domains. Indeed, in the SUMO conjugation pathway, the final step is catalysed by a limited number of SUMO E3 ligases^17^,^18^,^19^,^20^. These SUMO ligases, by facilitating the transfer of SUMO from the E2 conjugating enzyme (Ubc9) to a given substrate, increase specificity of SUMO conjugation. By using SUMO interacting motifs to recognize SUMO and by direct interactions with Ubc9, they engage the SUMO-loaded Ubc9 such that it is primed for transfer of SUMO to substrate^19^,^21^. The best-characterized SUMO E3 ligases are the Siz/PIAS (SP)-RING family and RanBP2 (ref. 22). Interestingly, a member of the PIAS protein family is encoded by the Su(var)2-10 locus in *Drosophila*^23^. In this work, we investigated whether Suv39h1 and Suv39h2, known to interact with HP1α (refs 24, 25), could function to enhance SUMO modification of HP1α. Using a combination of biochemical and cellular studies, we report that the Suv39h1 paralog enhances HP1α sumoylation. This capacity is independent from its H3K9 methyltransferase activity. In the N-terminal portion of the protein of key importance for this activity, we identify key residues for binding to Ubc9. Importantly, the same region promotes targeting of HP1α at pericentric domains when its binding to Ubc9 is intact. Our results unveil an unanticipated function for Suv39h1 that provides a missing link between the SUMO pathway and HP1α marking at pericentric heterochromatin.

## Results

### Intact Suv39h1 but not Suv39h2 enhances HP1α sumoylation

**Figure 1:**
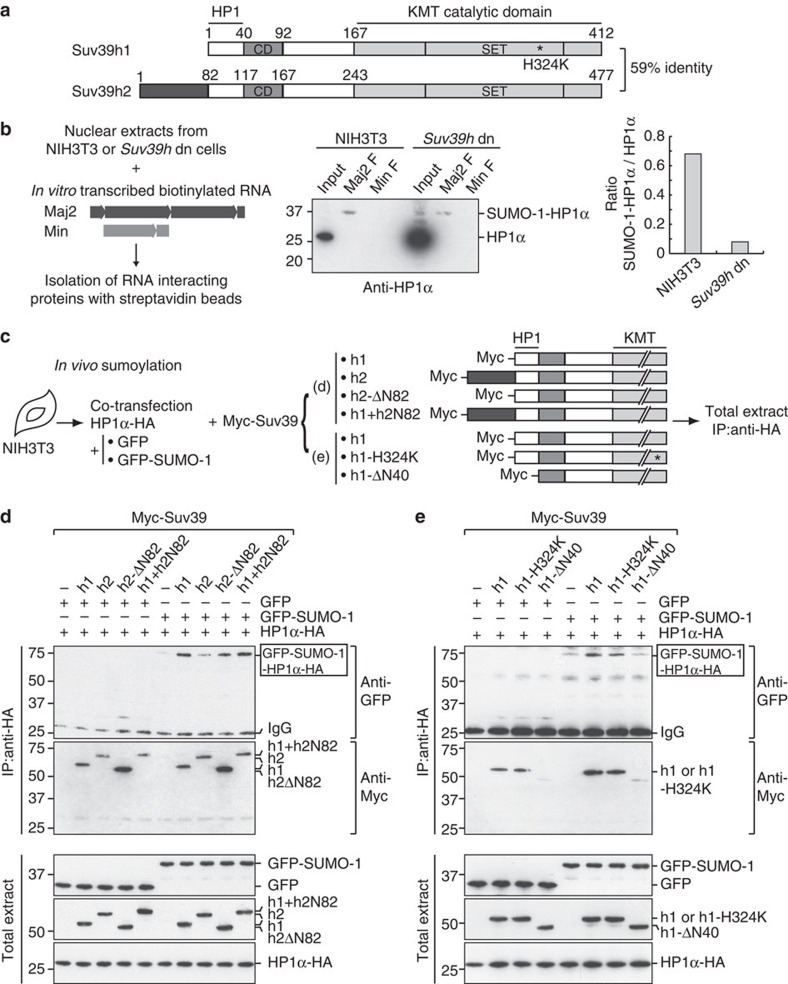
Suv39h1 enhances HP1α sumoylation *in vivo*. (**a**) Schematic representation of Suv39h1 and Suv39h2. (**b**) SUMO-1-HP1α retrieved from NIH3T3 or *Suv39h* double-null cells. We used nuclear extracts prepared in presence of *N*-ethylmaleimide (NEM) for RNA pull-down using forward (F) major (Maj2 F) or minor (Min F) RNAs as baits. Western blot analysis using anti-HP1α antibodies revealed endogenous sumoylated HP1α (SUMO-1-HP1α) and unmodified HP1α. Input is 5% of nuclear extracts. The histogram shows the ratio between SUMO-1-HP1α (bound to RNA) and unmodified HP1α obtained, respectively, for NIH3T3 and *Suv39h* double-null cells. (**c**) Experimental scheme. (**d**) *In vivo* HP1α sumoylation assay. After anti-HA immunoprecipitation, western blot analysis using anti-GFP antibodies revealed sumoylated HP1α-HA (GFP-SUMO-1-HP1α-HA, box). Western blot using anti-Myc antibodies indicated that the four Myc-tagged proteins coimmunoprecipitated with HP1α-HA. The expression of all transfected proteins assessed in total cell extracts is shown by western blot using anti-GFP, anti-Myc and anti-HA antibodies. IgG corresponds to the immunoglobulin light chain. (**e**) As in (**d**) with the mutant versions Suv39h1-H324K and Suv39h1-ΔN40.

We hypothesized that the two paralogs, Suv39h1 and Suv39h2, in mice represented candidates to enhance HP1α sumoylation. Comparison of their domain organization shows that beside a 82aa N-terminal extension in Suv39h2, they are highly similar (Fig. 1a). Using an RNA pull-down strategy described in ref. 16 that previously allowed us to successfully reveal SUMO-1-HP1α, we found that, in mouse embryonic fibroblasts (MEFs) derived from *Suv39h* double-null mice^4^, the proportion of SUMO-1-HP1α versus total HP1α dramatically decreased (Fig. 1b). This first indication in line with a loss of function prompted us to further explore a possible direct contribution of the Suv39h enzymes in promoting HP1α sumoylation *in vivo*. To further test this hypothesis, we performed a standard *in vivo* sumoylation assay in NIH3T3 cells (Fig. 1c). In this assay, after immunoprecipitation of HP1α-HA, sumoylation of HP1α is detected as a 70-kD band corresponding to GFP-SUMO-1-HP1α-HA. Introduction of the individual Myc-Suv39h paralogs to reach equal expression levels showed that only Suv39h1 could increase the levels of HP1α sumoylation, while Suv39h2 did not lead to any significant change (Fig. 1d and Supplementary Fig. 1). We verified that the two proteins co-immunoprecipitated with HP1α-HA at comparable level in these experiments. The expression of a truncated Suv39h2 lacking the first 82aa (Suv39h2ΔN82) enabled to detect HP1α sumoylation, suggesting that the extended N-terminus could possibly interfere with the ability to promote HP1α sumoylation. However, this interference only worked on the cognate Suv39h2, since a fusion comprising the first 82aa of Suv39h2 grafted onto the N-terminus of Suv39h1 (Suv39h1+h2N82) could not interfere with the ability of Suv39h1 to increase the levels of HP1α sumoylation. Altogether, these data demonstrate that Suv39h1, but not Suv39h2 unless truncated, can promote HP1α sumoylation *in vivo.* It is thus possible that Suv39h2 might exist in an autoinhibited state as reported recently for PARKIN (refs 26, 27). We next investigated whether this property in Suv39h1 can be separated from its H3K9 KMT activity and its interaction with HP1α. A H3K9 KMT catalytically dead mutant (Myc-Suv39h1-H324K)^3^ used in our assay led to enhanced amounts of sumoylated HP1α, in contrast to a truncated form lacking the HP1 binding domain (Myc-Suv39h1-ΔN40)^24^,^28^ (Fig. 1e and Supplementary Fig. 2). These data indicate that Suv39h1 promotes HP1α sumoylation regardless of its H3K9 KMT activity but absolutely requires an interaction with HP1α. Finally, given that *in vivo* assays could reflect indirect effects, such as the binding of a SUMO ligase activity to Suv39h1, we used an *in vitro* sumoylation assay with purified recombinant proteins. We could confirm that Suv39h1 on its own stimulated HP1α sumoylation *in vitro* in a dose-dependent manner (Supplementary Fig. 3). Furthermore, two other HP1α-binding partners, KAP1 (ref. 29) and p150 the large subunit of CAF-1 (ref. 30) known to have a link with SUMO (refs 31, 32) did not promote HP1α sumoylation, thus further underlining the specificity of this activity of Suv39h1.

### Suv39h1 interacts directly with Ubc9 via a 1–167 domain

**Figure 2:**
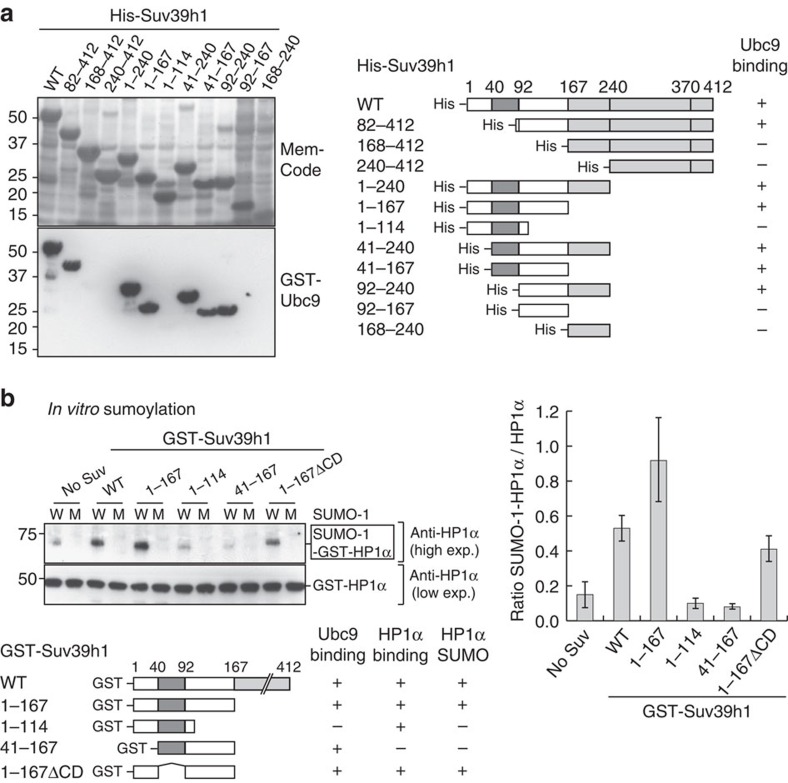
The 1–167 domain of Suv39h1 is responsible for promoting HP1α sumoylation. (**a**) Far-western blot using His-Suv39h1 full-length (WT) or truncated versions as baits and purified recombinant GST-Ubc9 as prey. We revealed bound GST-Ubc9 using anti-GST antibodies. Along each construct, interaction (+) or absence of interaction (−) with Ubc9 is indicated. (**b**) *In vitro* HP1α sumoylation assay. Top: western blot analysis of the sumoylation reaction mixture with anti-HP1α antibodies revealed sumoylated GST-HP1α (SUMO-1-GST-HP1α, box) and unmodified GST-HP1α. We performed quantitative analysis of this assay. The histogram shows the comparison of the ratio between SUMO-1-GST-HP1α and unmodified HP1α signal from western blot in the presence of Suv39h1-WT or truncated versions (Supplementary Fig. 5c). Error bars represent s.d. from three independent experiments. Bottom: results summary.

To further characterize the SUMO modification enhancing activity of Suv39h1, we thus examined whether Suv39h1 could bind directly to Ubc9 as found for the classical SUMO ligase enzymes^17^,^19^. Using GST pull-down and far-western assays, we found that Suv39h1 interacts directly with Ubc9 (Fig. 2a and Supplementary Fig. 4a,b). This is in line with classical SUMO ligases, but different from atypical SUMO ligase activities such as those associated with the SLX4 complex^33^ that only bind SUMO charged Ubc9. We then sought to define which critical domain in Suv39h1 is involved. We observed that only His-Suv39h1 truncation mutants containing the linker region (92-167) between the chromodomain (CD) and the KMT catalytic domain strongly interact with GST-Ubc9 (Fig. 2a and Supplementary Fig. 4b). We could further dissect the Ubc9 interaction domain within Suv39h1 to a region corresponding to aa114–140 (Supplementary Fig. 4c). Interestingly, we found that this 114–140 region is sumoylated *in vitro* (Supplementary Fig. 5a), consistent with a recent proteome-wide analysis in human cells that identified Suv39h1 as putative sumoylated proteins^34^. Furthermore, we found that Suv39h1 is a SUMO-1 interacting protein, where aa92-167 of Suv39h1 proved critical for this interaction (Supplementary Fig. 5b). Importantly, the 1–167 domain of Suv39h1 also mediates the interaction with HP1α through the first 40aa (refs 24, 28). Taken together, these data indicate that the 1–167 domain could confer on Suv39h1 the ability to enhance SUMO modification of HP1α consistent with Suv39h1 possessing SUMO E3 ligase. We then examined whether the 1–167 domain on its own could be sufficient to promote HP1α sumoylation *in vitro*. The addition of Suv39h1-1-167 clearly stimulated sumoylation of HP1α *in vitro* (Fig. 2b). Notably, deletion of the regions responsible for the interaction with Ubc9 (114-167) or HP1α (1-40) abolished HP1α sumoylation enhancement. Finally, we confirmed *in vivo* that the 1–167 domain of Suv39h1 promotes HP1α sumoylation (Supplementary Fig. 6). Collectively, these data demonstrate that the 1–167 domain is the key domain in Suv39h1 responsible for enhancing sumoylation and that it does not overlap with the H3K9 KMT domain (167-412). Of note, the 1–167 domain of Suv39h1 is distinct from the canonical RING-type or HECT-type E3 ligases and it has no substantial homology to any known SUMO E3 ligases. To formally determine if this represents a new type of SUMO E3 ligase, future work should focus on this 1–167 domain for detailed enzymatic and structural studies.

### Suv39h1 promotes HP1α *de novo* localization

We previously found that enhancing HP1α sumoylation could promote its *de novo* targeting to pericentric heterochromatin in the absence of Suv39h-dependent H3K9me3 (ref. 16). This ‘*de novo* HP1α localization assay' described in ref. 16 exploits *Suv39h* double-null cells with pericentric heterochromatin domains that have lost both HP1α and the H3K9me3 mark^4^,^35^. Using this assay, it was thus possible to test whether Suv39h1 acting solely by enhancing sumoylation of HP1α could promote *de novo* targeting of HP1α to pericentric domains. We thus introduced the H3K9 KMT catalytically dead mutant Myc-Suv39h1-H324K into the *Suv39h* double-null cells and monitored *de novo* pericentric localization of exogenous HP1α-HA (Supplementary Fig. 7a) and endogenous HP1α (Supplementary Fig. 7b). We found that in ∼11% of cells expressing Myc-Suv39h1-H324K, exogenous HP1α-HA and endogenous HP1α localized at pericentric domains lacking H3K9me3 (Supplementary Fig. 7a,b). To get an estimation of the expression levels of the exogenous proteins, we analysed protein levels by western blot on population of cells corresponding either to transfected *Suv39h* double-null MEF cells or in wild-type MEF cells (Supplementary Data). Since western blot analysis provides an average of the expression in the cells, we cannot formally exclude that at individual cell levels variation could occur. Thus, we conclude, that within the limits of our approach, the average expression level determined for the transfected cells remained close to the endogenous levels. Remarkably, these 11% of cells parallel the 10% of cells transfected with HP1α-Ubc9-HA that show the typical pericentric localization pattern in the absence of H3K9me3 (Supplementary Fig. 7a). It should be noted that both the fusion with Ubc9 (ref. 16), a means to bypass the requirement for a ligase, and the expression of the H3K9 KMT catalytically dead mutant (Myc-Suv39h1-H324K; Fig. 1e) increase levels of HP1α sumoylation *in vivo* and also lead to HP1α *de novo* localization at pericentric heterochromatin with similar efficiency. Notably, a H3K9 KMT catalytically dead mutant of Suv39h2 (Myc-Suv39h2-H398K) did not promote *de novo* localization of exogenous HP1α-HA at pericentric heterochromatin (Supplementary Fig. 8). This confirms that, except for the KMT activity, Suv39h2 cannot substitute for Suv39h1. Only the KMT catalytically dead Suv39h1 paralog (Suv39h1-H324K) can promote HP1α targeting to pericentric domains, in a manner that compares to an HP1α-Ubc9 fusion. Based on these data, we thus propose a new function for Suv39h1, independent of its H3K9 KMT activity, involved in *de novo* localization of HP1α at pericentric heterochromatin.

### TALE-mediated tethering of Suv39h to pericentric domains

**Figure 3:**
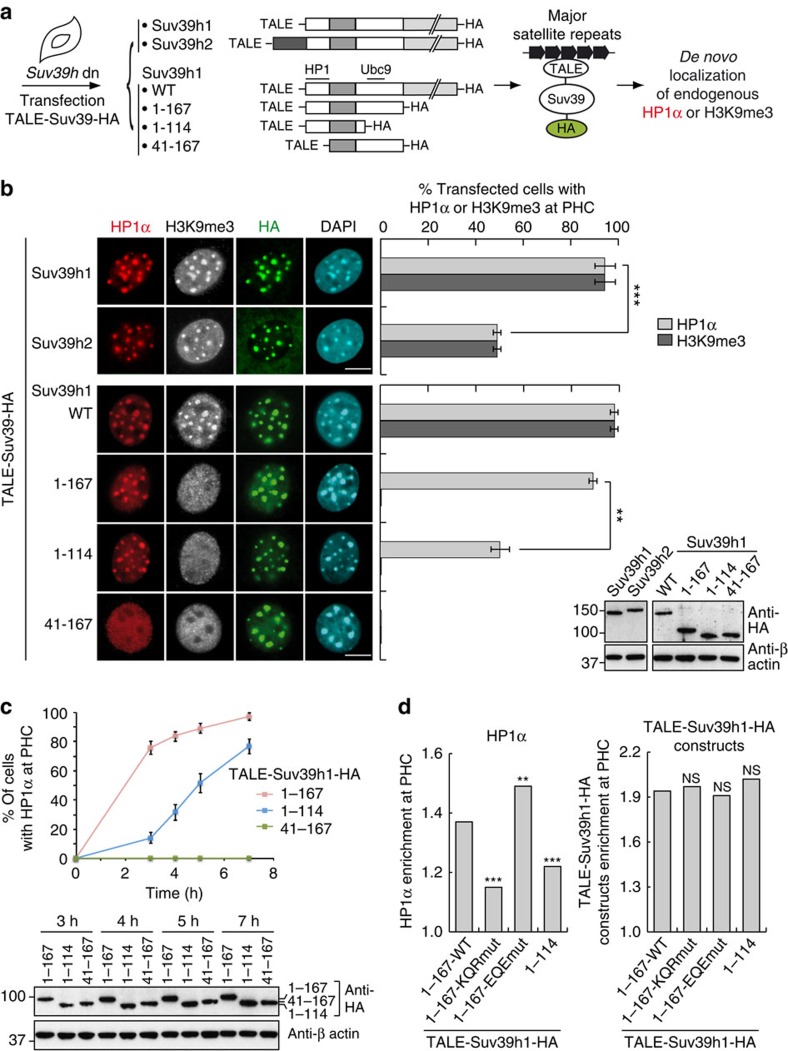
Tethering the 1–167 domain of Suv39h1 to pericentric heterochromatin accelerates *de novo* targeting of HP1α. (**a**) Experimental scheme. (**b**) *De novo* localization of endogenous HP1α (red) and H3K9me3 (white) in *Suv39h* double-null cells expressing TALE-Suv39h2, TALE-Suv39h1 or TALE-Suv39h1 domains tagged with HA analysed by immunofluorescence 5 h after transfection. Suv39-HA constructs are tethered to pericentric heterochromatin via TALE engineered to bind specifically to major satellite repeats in pericentric domains and revealed by HA staining (green). Scale bar, 10 μm. We examined 300 transfected cells and performed quantitative analysis of the percentage of transfected cells with endogenous HP1α or H3K9me3 enriched at pericentric heterochromatin domains (PHC). Error bars on the graph represent s.d. from three independent experiments. A comparison of transfected protein expression is shown on the Western blot. **P<0.01; ***P<0.001 (Student's *t*-test). Scale bar, 10 μm. (**c**) Time course analysis of the *de novo* localization of endogenous HP1α in *Suv39h* double-null cells transfected with TALE-Suv39h1 domains tagged with HA. The percentage of cells with HP1α enriched at pericentric heterochromatin domains (PHC) as a function of time after transfection is represented. Error bars indicate s.d. of four independent experiments (400 transfected cells counted in each condition). A comparison of transfected protein expression is shown on the western blot. (**d**) *De novo* localization of endogenous HP1α in *Suv39h* double-null cells transfected with TALE-Suv39h1-1-167-WT, TALE-SUV39h1-1-167-KQRmut, TALE-SUV39h1-1-167-EQEmut or TALE-Suv39h1-1-114 tagged with HA, 5 h post transfection. We performed quantitative analysis of endogenous HP1α (left) and TALE-Suv39h1-HA constructs (right) enrichment at PHC on more than 100 transfected cells for each condition from three independent experiments. NS, not significant; **P<0.01; ***P<0.001 (Student's *t*-test).

To directly address the potential role of Suv39h1 linked to the SUMO pathway in the ‘seeding step' for the *de novo* targeting of HP1α to pericentric domains, we decided to assess the capacity of the sole 1–167 domain of Suv39h1 as described above. However, our first attempt in *Suv39h* double-null cells showed that Myc-Suv39h1-1-167 did not even localize at pericentric domains (Supplementary Fig. 9a,b). We estimated by western blot, in our experimental conditions, how exogenous Myc-Suv39h1 expression compared with the endogenous Suv39h1 by quantifying the levels of Suv39h1 normalized to the proportion of transfected (expressing) cells (Supplementary Fig. 9c). Again, since western blot analysis provides an average of the expression in the cells, we cannot formally exclude that at individual cell levels variation could occur. Yet, within these limits the levels were comparable. In fact, we found that all truncated form (N- or C-terminal) of Suv39h1 failed to localize *de novo* at pericentric heterochromatin, indicating that the full integrity of the protein is absolutely necessary for its targeting (Supplementary Fig. 9b). Without this localization, we could not monitor a *de novo* targeting of HP1α arguing for the necessity of Suv39h1 and the truncated versions to be at the site to sumoylate HP1α at pericentric domains for its effective targeting/retention required for the seeding step. Considering the importance of a local action, we thus decided to artificially tether the 1–167 domain of Suv39h1 to pericentric domain to be able to assess HP1α *de novo* localization to pericentric heterochromatin in *Suv39h* double-null cells. For this, we used transcription activator-like effectors (TALE) engineered to bind specifically to the major satellite DNA repeats found at pericentric heterochromatin^36^. We first fused this TALE with full-length Suv39h1 and Suv39h2 (Fig. 3a). After transfection in *Suv39h* double-null cells, we verified by immunofluorescence that they accumulated at pericentric domains and could restore H3K9me3 enrichment (Fig. 3b). As above, we also verified expression levels of exogenous TALE-Suv39h1 compared with endogenous Suv39h1 (Supplementary Fig. 9c). Intriguingly, pericentric localization of endogenous HP1α was less efficient in the presence of tethered Suv39h2-HA compared with tethered Suv39h1-HA at early times after transfection (Fig. 3b and Supplementary Fig. 10). This argues for an additional role for Suv39h1 not fulfilled by the Suv39h2, in line with the inability of Suv39h2 full-length to promote HP1α sumoylation (Fig. 1d). This also underlines the unique specificity of Suv39h1 to promote HP1α targeting to pericentric domains independently of its KMT activity.

### The sole 1–167 domain accelerates HP1α *de novo* targeting

We next used TALE fusions to investigate the impact of the 1–167 domain of Suv39h1 supporting the sumoylation of HP1α. We generated TALE fusions of the entire 1–167 domain (Suv39h1-1-167-HA) and two deleted 1–167 constructs, one unable to interact with Ubc9 (Suv39h1-1-114-HA), and one unable to interact with HP1α (Suv39h1-41-167-HA). These two latter constructs proved inefficient in promoting HP1α sumoylation (Fig. 2b). All TALE-Suv39h1-HA constructs could accumulate at chromocenters after transfection in *Suv39h* double-null cells (Fig. 3b). Importantly, when tethering Suv39h1-1-167-HA, even in the absence of detectable H3K9me3, we could readily detect endogenous HP1α *de novo* localization at pericentric domains with an efficiency of 89%. It dropped to 50% when tethering Suv39h1-1-114-HA, as assessed 5 h post transfection. In contrast, no detectable HP1α localization was observed in the presence of tethered Suv39h1-41-167-HA. Thus, while tethering constructs of Suv39h1 that contains the HP1α binding domain (1–40) is sufficient to detect HP1α accumulation at pericentric heterochromatin, the additional presence of the Ubc9 binding domain (114–140) is absolutely essential to enhance the *de novo* localization of HP1α at these regions. To rule out the possibility that increased HP1α *de novo* localization mediated by the 1–167 domain could simply occur via stronger interactions with HP1α compared with the 1–114 domain, we further monitored the binding of HP1α to the 1–167 or the 1-114 domain of Suv39h1. Using either far-western assays or AlphaScreen technology, we could not observe differences in the capacity of the two domains of Suv39h1 to interact with HP1α (Supplementary Fig. 11a,b). Moreover, we found that TALE-Suv39h1-1-167-HA and TALE-Suv39h1-1-114-HA co-immunoprecipitated equally endogenous HP1α (Supplementary Fig. 11c). Thus a major difference in HP1 binding could not explain our findings but rather confirms the hypothesis that the ability of Suv39h1 to interact with Ubc9 is a critical parameter to boost targeting of HP1α. We thus focused on the two following constructs, TALE-Suv39h1-1-167-HA and TALE-Suv39h1-1-114-HA, to monitor the kinetics of endogenous HP1α appearance at pericentric heterochromatin following their transfection in the *Suv39h* double-null cells (Fig. 3c and Supplementary Fig. 11d). At early time points, the appearance of HP1α at pericentric domains proved most efficient for Suv39h1-1-167 compared with Suv39h1-1-114 construct. This was achieved while expression of the HA-corresponding tagged proteins had reached comparable levels throughout the time-course (Fig. 3c) and showed comparable levels of accumulation at pericentric domains indicating that HP1α localization at pericentric heterochromatin cannot be simply due to high levels of Suv39H1 expression (Supplementary Fig. 11d). Taken together, these data demonstrate that the presence of the Ubc9 binding domain within the 1–167 domain of Suv39h1 to pericentric heterochromatin is key to accelerate HP1α targeting to these domains.

### Identification of specific residues in the 1–167 domain

To delineate which amino acids within Suv39h1-1–167 were most critical for the interaction with Ubc9, we generated four triple point alanine mutants based on amino acids conservation in vertebrate species and in Drosophila (Supplementary Fig. 12a,b). When compared with the wild-type version, we found that the KQR mutation reduced both the binding of the 1–167 domain of Suv39h1 to Ubc9 (Supplementary Fig. 12b) and HP1α sumoylation *in vivo* (Supplementary Fig. 13). In contrast, the EQE mutation rather showed a slight increase for Ubc9 interaction. We then transfected TALE-1-167-KQRmut-HA in *Suv39h* double-null cells to assess its capacity to promote HP1α targeting to pericentric domains. Remarkably, the 1–167-KQRmut, similar to the 1–114 domain, performed less well than the 1–167-WT or 1-167-EQEmut (Fig. 3d and Supplementary Fig. 14). Since all the TALE-constructs accumulated at pericentric domains in a similar way, the possibility of a distinct capacity in their targeting or a simple effect of expression levels can be discarded. Of note, we realized that the 1-167-EQEmut was even more potent that the WT in promoting HP1α enrichment. This enhanced capacity parallels its increased ability to bind to Ubc9. Overall, our data underline the importance of a functional Ubc9 binding domain within Suv39h1 involving specific conserved amino acids to promote HP1α sumoylation and its targeting to pericentric heterochromatin.

## Discussion

**Figure 4:**
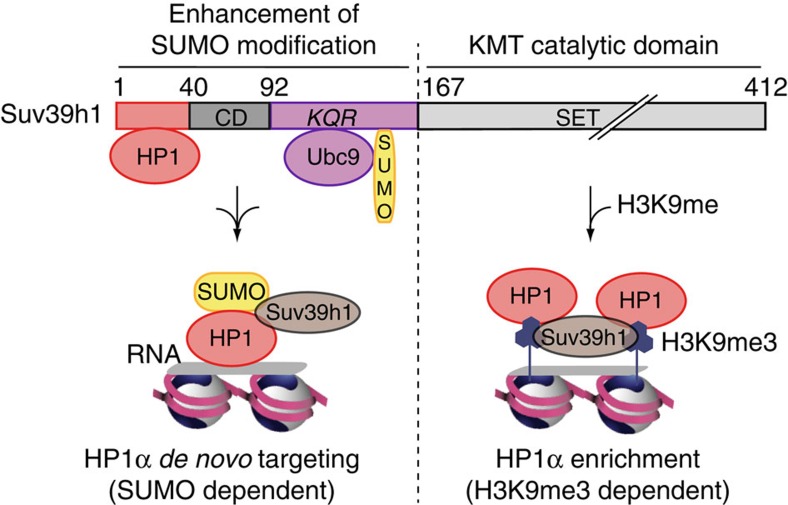
Model for a dual function of Suv39h1. The two functional domains in Suv39h1 highlighting the interaction with HP1α, Ubc9 and SUMO are depicted. During *de novo* HP1α targeting to pericentric heterochromatin, Suv39h1 can enhance HP1α sumoylation and accelerate its recruitment. During HP1α enrichment, Suv39h1 would function as KMT leading to H3K9 trimethylation thereby providing binding sites for HP1α allowing its accumulation/retention. While the two activities are associated with distinct subdomains, they may not necessarily function in an exclusive manner and rather provide a means to integrate a metabolic sensing to the specific targeting of a nuclear domain.

While we had previously learnt that HP1α sumoylation was key for its interaction with RNA and its *de novo* targeting to pericentric heterochromatin^16^, how this modification on HP1α was imposed and controlled remained a mystery. Furthermore, considering the recent connection between chromatin, histone modifiers and Ubc9 (ref. 37), the issue became particularly intriguing. Here, not only we identify a new control on the HP1α sumoylation, but most remarkably it involves a major histone-modifying enzyme with a known function in regulating chromatin state through methylation of histone, a modification linked to cellular metabolism^38^. This unexpected function for Suv39h1 in addition to its KMT activity as a constitutive property contrasts with the necessity to truncate the paralog Suv39h2 to unveil the same capacity. The distinct properties of the two Suv39h paralogs open up new ways to consider the connections between Suv39h and heterochromatin organization. Importantly, two regions in the Suv39h1 protein can be distinguished, with the 1–167 domain containing the Ubc9 binding motifs to enhance sumoylation and the 167–412 domain for the H3K9me3 KMT activity. While the two activities can be separated functionally, they may not necessarily function in an exclusive manner and rather help to connect specific nuclear organization to particular metabolic sensing. We propose a working model to account for this dual role of Suv39h1 in the *de novo* localization of HP1α and pericentric heterochromatin formation (Fig. 4). First, Suv39h1, through its capacity to promote HP1α sumoylation, would accelerate its recruitment through the specific association between SUMO-1-HP1α and major RNAs. Second, Suv39h1 with its KMT activity that trimethylates H3K9 would then generate anchoring sites to ensure a controlled maintenance of HP1α at pericentric domains^3^,^4^. This is somehow reminiscent of the dual modifications in facultative heterochromatin that are controlled by the Polycomb complex with H3K27me3 and H2A ubiquitylation^39^. In this scheme, it is tempting to speculate that the activity of Suv39h1 to enhance SUMO modification could be most critical when major chromatin rearrangements and *de novo* establishment of pericentric domains are needed as observed during early development^40^,^41^ or lymphocytes activation (unpublished results in collaboration with S. Amigorena), or other changes associated with metabolic stress and rewiring. The specific expression of Suv39h1, but not Suv39h2, during early mouse development^15^ points to interesting avenues to explore. Thus, this unique property of Suv39h1 can have a profound impact of physiological relevance. Indeed, Suv39h1 as the first enzyme promoting sumoylation identified at pericentric heterochromatin domains could also act on other components. Precedent for this in DNA repair^42^ showed that the recruitment of specific SUMO ligases at particular places often helps to target an entire group of functionally and physically connected proteins to build-up complex nuclear assemblies^21^. Suv39h1 could represent a major orchestrating activity with a broader impact on constitutive heterochromatin that one could have initially thought based only on the KTM activity. Indeed, many proteins linked to pericentric heterochromatin are known to be sumoylated, including KAP1 (ref. 31) and MeCP2 (ref. 43). Furthermore, some of them are even direct Suv39h1-interacting proteins, such as HDAC1 (refs 44, 45), Dnmt1 (refs 46, 47) and MBD1 (refs 48, 49). Finally, the conservation of Ubc9 binding and the link with the SUMO pathway deserves to be explored in *S. pombe* and *Drosophila* to deepen our understanding of the basic properties linked to heterochromatin establishment and propagation. Clearly, the recent finding revealing Ubc9 as a major repressor of iPS cell formation further underlines the importance of SUMO pathway in the context of somatic cell identity^37^. Future work should address these issues. While we have concentrated here on sumoylation, desumoylation as another means to regulate the steady-state achieved is equally important. In this respect, the SUMO protease SENP7 localized at pericentric heterochromatin^50^ will be a likely candidate to consider, in particular given its ability to bind to HP1 proteins^51^.

In conclusion, the link of Suv39h1 with the SUMO pathway discovered in this work provides a new conceptual framework for the dynamics and the stability of constitutive heterochromatin, paving the way for exciting work in the field of nuclear organization and its role during normal development and pathological situations.

## Methods

### Cell culture and extracts

We cultured *Suv39h* double-null MEFs (provided by T. Jenuwein)^4^ and NIH3T3 cells (ATCC #CRL-1658) in Dulbecco's modified Eagle's medium (Invitrogen) supplemented with 10% foetal calf serum (Eurobio), 100 U ml^−1^ penicillin and 100 μg ml^−1^ streptomycin (Invitrogen) at 37 °C and 5% CO_2_. We tested them for mycoplasma contamination (Mycoplasma PCR ELISA, Sigma). We transfected MEFs and NIH3T3 cells with Nucleofector Kit 2 (Amaxa) and Lipofectamine 2000 (Invitrogen) respectively, according to manufacturer's instructions. We prepared total cell extracts by resuspending cells in lysis buffer (50 mM Tris-HCl pH 7.5, 150 mM NaCl, 5 mM EDTA, 15 mM MgCl_2_, 1% Nonidet P-40 and 0.75% sodium deoxycholate) supplemented with protease and phosphatase inhibitors, and 20 mM *N*-ethylmaleimide (Sigma).

### Plasmids

We obtained GST-Suv39h1 full-length and 1–114 from K. Yamamoto^28^, GST-KAP1 from F. Rauscher^31^, GST-HP1α from the late R. Losson^52^, His-HP1α from S.M. Jang, GST-Ubc9 from M. Dasso, GST-SUMO-1 from G. Gill^53^, GFP-SUMO-1 from R. Hay, Myc-Suv39h1 full-length and truncated versions (Δchromo, ΔN89, Nchromo, ΔSET, cysSET used in Supplementary Fig. 9) from T. Jenuwein^24^. His-p150, HP1α-HA and HP1α-Ubc9-HA were described previously^16^,^54^. We generated GST-Suv39h1-1-167, 1-140, 41-167 and 1-167ΔCD by subcloning of full-length GST-Suv39h1. We obtained His-Suv39h1 by cloning full-length Suv39h1 (gift of K. Yamamoto) into pET-30a vector (Novagen) and truncation constructs of His-Suv39h1 by subcloning of full-length His-Suv39h1. To create Myc-Suv39h1-ΔN40, Myc-Suv39h1-1-167, Myc-Suv39h1-1-114 and Myc-Suv39h1-41-167, we made truncation constructs from full-length Myc-Suv39h1 (gift of T. Jenuwein). We generated Myc-Suv39h1-H324K point mutant using the Quick Change Site-Directed Mutagenesis Kit (Stratagene). We obtained Myc-Suv39h2 by replacing Suv39h1 cDNA of the Myc-Suv39h1 vector with mouse Suv39h2 cDNA^12^ (accession no. AF149205). We made Myc-Suv39h2-ΔN82 by subcloning from full-length Myc-Suv39h2. We obtained Myc-Suv39h1+h2N82 construct by inserting the first 82 amino acids of Suv39h2 at the N terminus of the Myc-Suv39h1 plasmid. To generate TALE-Suv39h2-HA, TALE-Suv39h1-WT-HA, TALE-Suv39h1-1-167-HA, TALE-Suv39h1-1-114-HA and TALE-Suv39h1-41-167-HA, we replaced the mClover of the pTALYM3B15 plasmid (obtained from Addgene #47878, ref. 36) by Suv39h2, full-length Suv39h1, 1–167, 1–114 and 41–167 fused to a HA-tag in the C-terminus, respectively. The point mutations in the Ubc9 binding domain of Suv39h1 were generated from Myc-Suv39h1, His-Suv39h1-1-167 and TALE-Suv39h1-1-167-HA by site directed mutagenesis (GenScript). All constructs were verified by sequencing.

### Antibodies and immunoprecipitations

We used mouse monoclonal anti-HP1α (2HP-1H5-AS for immunofluorescence and 2HP-2G9-AS for Western blot, Euromedex; 1:1,000), rabbit polyclonal anti-HP1α (#H2164, Sigma; 1:1,000), rabbit monoclonal anti-HP1α (#H2623, Cell Signaling; 1:1,000), mouse monoclonal anti-GFP (#11814460001, Roche; 1:1,000), rat monoclonal anti-HA (#1867423, Roche; 1:2,000 for western blot and 1:1,000 for immunofluorescence), mouse monoclonal anti-Myc (ab32, Abcam; 1:1,000), rabbit polyclonal anti-H3K9me3 (#39765, Active Motif; 1:500), rabbit monoclonal anti-Suv39h1 (#8729, Cell Signaling, 1:1,000), rabbit polyclonal anti-GST (ab9085, Abcam; 1:1,000) and mouse monoclonal anti-β actin (#A5441 Sigma; 1:20,000). We performed anti-HA immunoprecipitations by incubating total cell extracts corresponding to 4 × 10^6^ cells with 40 μl of monoclonal anti-HA agarose-conjugated beads (Roche) for 2 h at 4 °C. After washing the beads with lysis buffer, we eluted the immunocomplexes with SDS-polyacrylamide gel electrophoresis (PAGE) loading buffer^16^.

### *In vitro* sumoylation assay and western blot

We produced recombinant proteins in *Escherichia coli* BL21 (DE3) corresponding to GST-HP1α, GST-KAP1, His-p150 and GST-Suv39h1 full-length protein and truncation fragments. Following SDS-PAGE, we analysed these proteins by gel staining (Imperial Protein Stain, Pierce). We used the recombinant proteins in *in vitro* sumoylation reactions using the SUMOlink kits (Active Motif) according to the manufacturer's instructions, except that we diluted 1:4 the E2 conjugating enzyme Ubc9. Unless stated otherwise, we added 60 ng of GST-Suv39h1 full-length or truncated versions, GST-KAP1 or His-p150. We stopped the reactions by addition of 2 × SDS-PAGE loading buffer. Reaction mixture (25%) was run on a 4–12% Bis-Tris NuPAGE gel (Invitrogen) and transferred onto nitrocellulose membranes (Protran). We probed sumoylated and unmodified GST-HP1α using mouse monoclonal anti-HP1α antibodies and horse radish peroxidase-conjugated secondary antibodies (Jackson Immunoresearch). Before the visualization of proteins using the Super Signal West Pico chemiluminescence substrates (Pierce), we cut the membrane just above the protein marker 50 kDa and performed two different exposures: high to enable the detection of SUMO-1-GST- HP1α and a low to visualize unmodified GST-HP1α (see Supplementary Fig. 3). Western blots with high and low exposures corresponding to this cut membrane are shown in Fig. 2b. We quantified SUMO-1-HP1α and HP1α from western blots with an ImageQuant LAS 4000 mini system and the ImageQuant software (GE Healthcare). The western blot in Fig. 2a is not cropped. Uncropped western blots of Figs 1 and 3 are shown in Supplementary Figs 15–19.

### Centromeric RNA pull-down

We prepared nuclear extracts from NIH3T3 cells or Suv39h double-null MEFs and performed RNA pull-down using published procedures^16^ in the presence of 20 mM *N*-ethylmaleimide (Sigma).

### GST pull-down

We expressed GST and GST-Ubc9 in *Escherichia coli* BL21 (DE3) and immobilized proteins on Glutathione Sepharose 4B (GE Healthcare). We performed GST pull-down by mixing GST or GST-Ubc9 beads with total extract of NIH3T3 cells expressing Myc-Suv39h1. We incubated for 2 h at 4 °C on a rotating wheel in binding buffer (20 mM Tris-HCl pH 8, 200 mM NaCl, 2 mM EDTA, 0.5% (V/V) Nonidet P-40) supplemented with proteases inhibitors. After five washes with binding buffer, the proteins were recovered by boiling the beads in SDS–PAGE loading buffer and were analysed by western blot.

### Far western blot

We resolved lysates of *Escherichia coli* BL21 (DE3) expressing His-Suv39h1 full-length and truncation fragments by SDS–PAGE and transferred them onto nitrocellulose membranes (Protran). Proteins on membranes were denatured/renatured and incubated with purified recombinant GST-Ubc9, GST-SUMO-1, GST-HP1α and GST as described in ref. 55. We detected bound GST-Ubc9, GST-SUMO-1 and GST-HP1α using anti-GST antibodies.

### AlphaScreen assays

We applied the AlphaScreen technology (amplified luminescent proximity homogeneous assay, PerkinElmer) to perform saturation binding assays using fixed concentrations of GST-Suv39h1 truncated versions with variable His-HP1α concentrations. We produced recombinant proteins in *Escherichia coli* BL21 (DE3) corresponding to His-HP1α, GST-Suv39h1-1-167, GST-Suv39h1-1-114 and GST-Suv39h1-41-167 constructs. For our analysis in 96-well plate, we incubated His-HP1α proteins with each of the GST-Suv39h1 truncated forms for 2 h at room temperature in 50 μl of assay buffer (20 mM Tris pH 7,5; 100 mM NaCl; 2 mM MgCl_2_; 1 mM DTT; 0,05% BSA; and 0.1% Tween). Then we added the acceptor beads (Nickel-Chelate acceptor) and the donor beads (Glutathione donor) at a final concentration of 20 μg ml^−1^ for a second incubation of 2 h. For Alpha counts acquisition, we used an EnSpire plate reader (PerkinElmer).

### Immunofluorescence

We processed cells for immunostaining as described in ref. 35. We detected proteins of interest using the appropriate primary and secondary antibodies conjugated to Alexa-Fluor 488, 594 and 647 (Invitrogen). We used a Zeiss Imager Z1 epifluorescence microscope piloted with Metamorph software, an × 63 oil objective lens and an HQ2 CoolSnap camera (Photometrics) for image acquisition. We obtained percentages of cells with HP1α or H3K9me3 localized at pericentric heterochromatin by counting more than 100 cells in each experiment. To quantify the enrichment of endogenous HP1α or TALE-Suv39h1-HA constructs at pericentric heterochromatin domains, we used the three-dimensional-fluorescence intensity enrichment at domains ImageJ macro^56^ from Z-stacks images acquired with a Deltavision system equipped with an inverted Olympus IX71 microscope and a × 100 objective lens (Supplementary Fig. 11d) or with a Zeiss Z1 epifluorescence microscope and a × 63 objective lens (Fig. 3d). The procedure first uses the 4,6-diamidino-2-phenylindole stacks to segment the nucleus and the pericentric domains to define three-dimensional masks that correspond to these regions. These masks are then applied to the fluorescent channels corresponding to HP1α and TALE-Suv39h1-HA constructs to quantify their fluorescence intensity detected within and outside pericentric domains. This fluorescence intensity is then normalized to the size of the domains and the nucleus. Enrichment for HP1α and TALE-Suv39h1-HA constructs is obtained by the ratio of these normalized fluorescence intensity within and outside of the domains. A value equal/close to 1 represents no enrichment (equal normalized fluorescence within and outside the domains) whereas increasing values quantify increasing enrichment at the pericentric domains. We performed the quantification on at least 100 nuclei for each condition. For all studies, we performed at least three independent experiments.

### Data availability

The authors declare that the data supporting the findings of this study are available within the article and its Supplementary Information files, or from the authors on reasonable request.

## Additional information

**How to cite this article**: Maison, C. *et al*. The methyltransferase Suv39h1 links the SUMO pathway to HP1α marking at pericentric heterochromatin. *Nat. Commun.* 7:12224 doi: 10.1038/ncomms12224 (2015).

## Supplementary Material

Supplementary Information Supplementary Figures 1-19 and Supplementary References.

Peer Review File
