# Supplementary material for: The methyltransferase Suv39h1 links the SUMO pathway to HP1α marking at pericentric heterochromatin
Source: Nat Commun. 2016 Jul 18;7:12224. doi: 10.1038/ncomms12224 (PMC4960310; doi:10.1038/ncomms12224)
Supplement: Supplementary Information — Supplementary Figures 1-19 and Supplementary References. [file ncomms12224-s1.pdf]

SUPPLEMENTARY INFORMATION

Supplementary figures

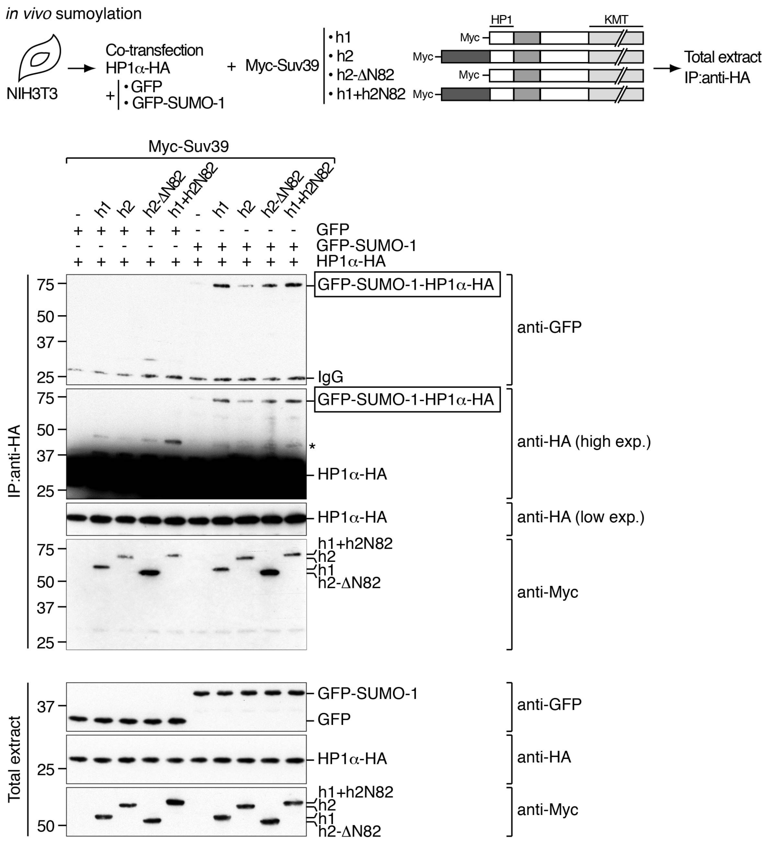

Supplementary Figure 1: Suv39h1, but not Suv39h2, promotes HP1α sumoylation *in vivo*.
In vivo HP1α sumoylation assay. Top: experimental scheme. Middle: we transfected NIH3T3 cells with HP1α-HA, GFP, GFP-SUMO-1, Myc-Suv39h1, Myc-Suv39h2, Myc-Suv39h2-ΔN82 or Myc-Suv39h1+h2N82 as indicated. We performed anti-HA immunoprecipitation from total extracts. Western blot analysis using anti-GFP and anti-HA antibodies revealed sumoylated HP1α-HA (GFP-SUMO-1-HP1α-HA, box) and unmodified HP1α-HA. Western blot using anti-Myc antibodies indicated that the four Myc-tagged proteins coimmunoprecipitated with HP1α-HA. Note that more Myc-Suv39h2-ΔN82 coimmunoprecipitated with HP1α-HA compared to wild type Suv39h2. IgG corresponds to the immunoglobulin light chain. The asterisk indicates unspecific bands. Bottom: We verified the expression levels of all transfected proteins in total cell extracts by Western blot using anti-GFP, anti-HA and anti-Myc antibodies.

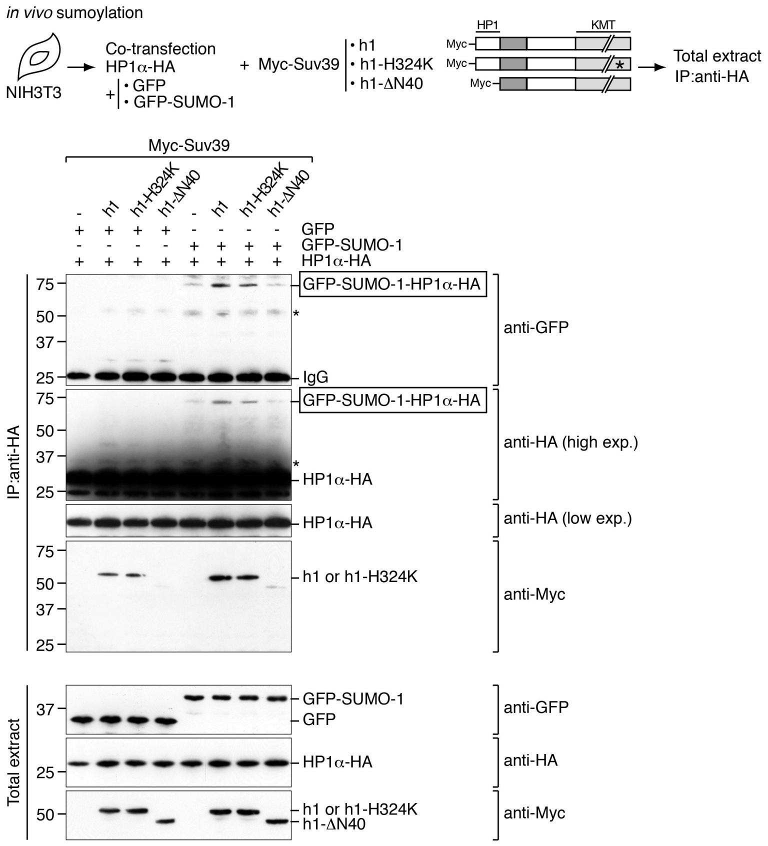

Supplementary Figure 2: Suv39h1 enhances HP1α sumoylation *in vivo* regardless of its H3K9 KMT activity.

In vivo HP1α sumoylation assay. Top: experimental scheme. Middle: we transfected NIH3T3 cells with HP1α-HA, GFP, GFP-SUMO-1, Myc-Suv39h1, Myc-Suv39h1-ΔN40 or Myc-Suv39h1-H324K as indicated. We performed anti-HA immunoprecipitation from total extracts. Western blot analysis using anti-GFP and anti-HA antibodies revealed sumoylated HP1α-HA (GFP-SUMO-1-HP1α-HA, box) and unmodified HP1α-HA. Western blot using anti-Myc antibodies indicated that Myc-Suv39h1 and Myc-Suv39h1-H324K but not with Myc-Suv39h1-ΔN40 coimmunoprecipitated with HP1α-HA. IgG corresponds to the immunoglobulin light chain. Asterisks indicate unspecific bands. Bottom: We verified the expression levels of all transfected proteins in total cell extracts by Western blot using anti-GFP, anti-HA and anti-Myc antibodies.

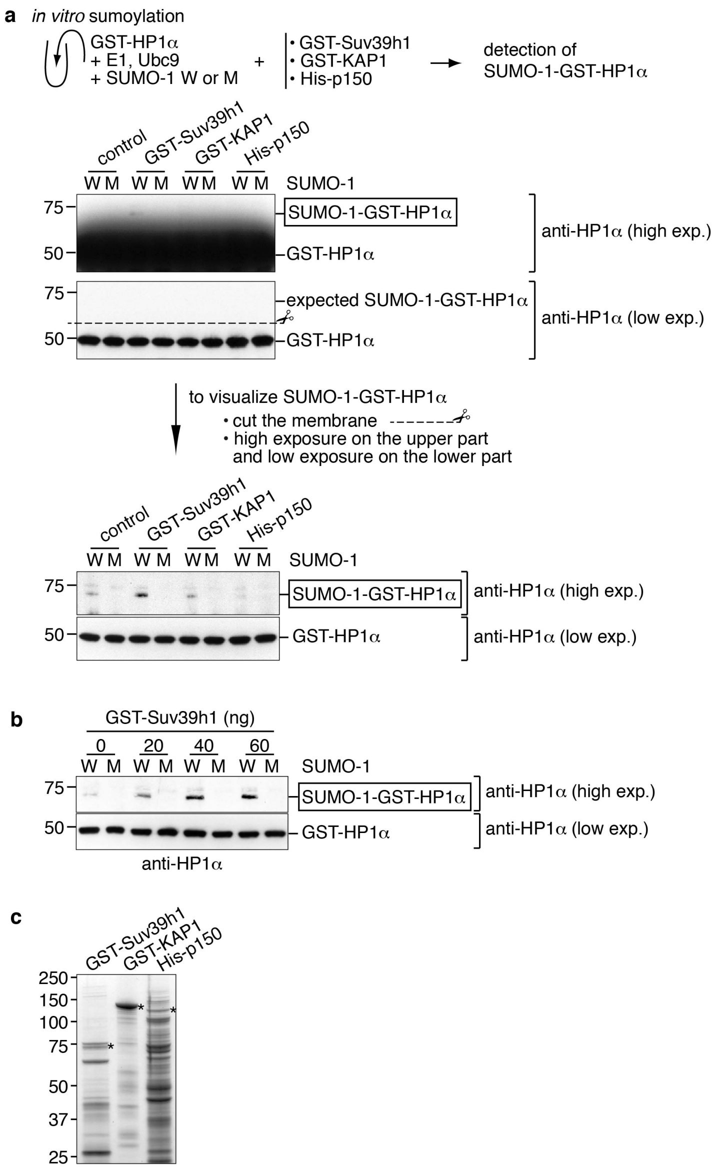

Supplementary Figure 3: Suv39h1 enhances HP1α sumoylation *in vitro*.

a. *In vitro* HP1α sumoylation assay. Top: experimental scheme. Equal amounts (60 ng) of HP1α partners (Suv39h1, KAP1 and p150) were tested for their ability to stimulate purified GST- HP1α sumoylation. Bottom: Analysis of the sumoylation reaction mixture by Western blotting using anti-HP1α antibodies revealed the positions of sumoylated GST-HP1α (SUMO-1-GST-HP1α, box) and unmodified GST-HP1α. Low exposure allows a clear detection of unmodified GST-HP1α but not of SUMO-1- GST-HP1α. High exposure permits detection of SUMO-1- GST-HP1α, but most of the signal is obscured by the high signal of the GST-HP1α below. Therefore, to optimize the detection of SUMO-1-GST-HP1α, we cut the membrane (scissors) above the protein marker 50 kDa before the detection step of the Western blotting procedure. We then performed a high exposure on the upper part that enabled the visualization of SUMO-1-GST-HP1α and a low exposure on the lower part that enabled the visualization of unmodified GST-HP1α. **b**. Assay of Suv39h1 in stimulating HP1α sumoylation *in vitro*. Western blot analysis was performed as in **a**. **c.** Analysis of recombinant GST-Suv39h1, GST-KAP1 and His-p150 used in sumoylation reactions by Imperial Protein staining.

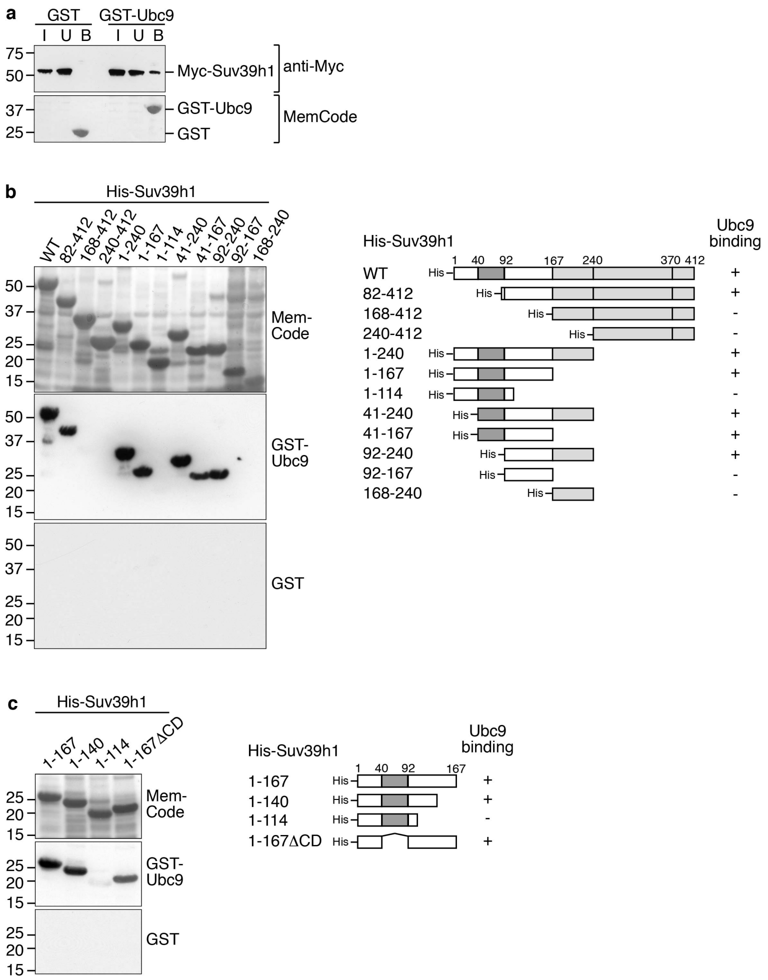

Supplementary Figure 4: Suv39h1 binds directly to Ubc9.

a. GST pull down assay. We incubated total extract of NIH3T3 cells expressing Myc-Suv39h1 with GST-Ubc9 or GST beads. We monitored the presence of Suv39h1 in the input (I, 10%), unbound (U) and bound (B) fractions by Western blot using anti-Myc antibodies. Memcode staining of the membrane revealed the similar amounts of GST-Ubc9 and GST used. **b**. Far-western blot using His-Suv39h1 full-length (WT) or truncated versions as baits and purified recombinant GST-Ubc9 or GST control as preys. We revealed interacting GST-Ubc9 using anti-GST antibodies. Along each construct, interaction (+) or absence of interaction (-) with Ubc9 is indicated. Note that the 92-167 domain alone failed to bind to Ubc9, suggesting that flanking amino acids might be required in this assay either for the interaction between Suv39h1 and Ubc9 or for the proper folding of the 92-167 truncated form necessary for the interaction with Ubc9. **c**. Far-western blot as above. We found that the Ubc9 interaction domain on Suv39h1 could be refined to aa114-140.

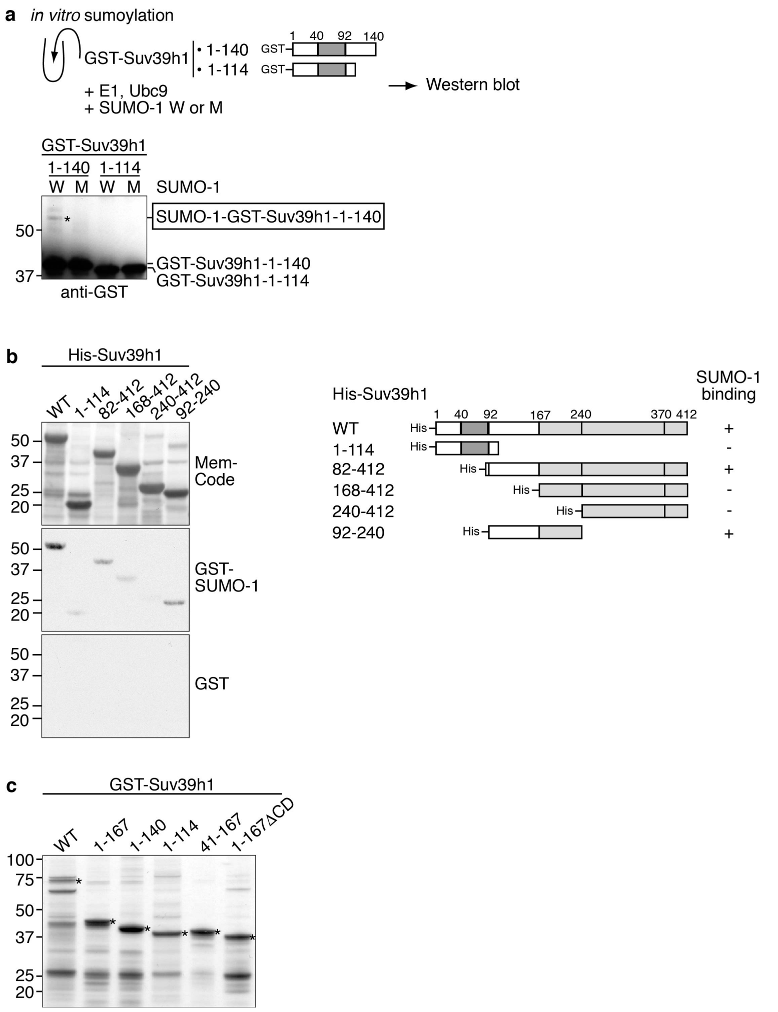

Supplementary Figure 5: Suv39h1 binds directly to SUMO-1.

a. *In vitro* Suv39h1 sumoylation assay with series of truncated versions. Top: experimental scheme. Bottom: Western blot analysis of the sumoylation reaction mixture with anti-GST antibodies revealed sumoylated GST-Suv39h1-1-140 (SUMO-1-GST-Suv39h1-1-140, box) marked by an asterisk and unmodified GST-Suv39h1-1-140 or GST-Suv39h1-1-114. **b**. Far-western blot using His-Suv39h1 full-length (WT) or truncated versions as baits and purified recombinant GST-SUMO-1 or GST control as preys. We revealed bound GST-SUMO-1 using anti-GST antibodies. Along each construct, interaction (+) or absence of interaction (-) with SUMO-1 is indicated. **c**. We analyzed by Imperial Protein staining recombinant GST-Suv39h1 full-length (WT) or truncated versions used in *in vitro* sumoylation reactions showed in Fig. 2b. Asterisks indicate the recombinant proteins.

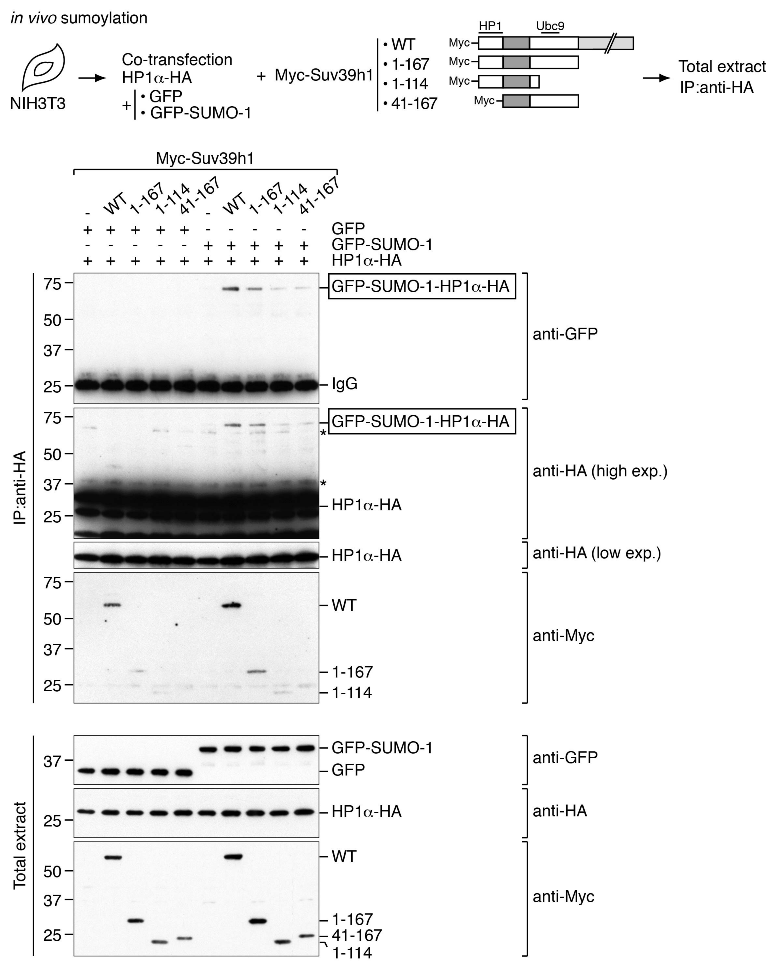

Supplementary Figure 6: The 1-167 domain of Suv39h1 promotes HP1α sumoylation *in vivo*. *In vivo* HP1α sumoylation assay. Top: experimental scheme. Middle: we transfected NIH3T3 cells with HP1α-HA, GFP, GFP-SUMO-1, Myc-Suv39h1-WT, Suv39h1-1-167, Suv39h1-1-114 or Suv39h1-41-167 as indicated. We performed anti-HA immunoprecipitation from total extracts. Western blot analysis using anti-GFP and anti-HA antibodies revealed sumoylated HP1α-HA (GFP-SUMO-1-HP1α-HA, box) and unmodified HP1α-HA. Western blot using anti-Myc antibodies indicated that Myc-Suv39h1-WT and Myc-Suv39h1-1-167 coimmunoprecipitated with HP1α-HA. IgG corresponds to the immunoglobulin light chain. Asterisks indicate unspecific bands. Bottom: We verified the expression levels of all transfected proteins in total cell extracts by Western blot using anti-GFP, anti-HA and anti-Myc antibodies.

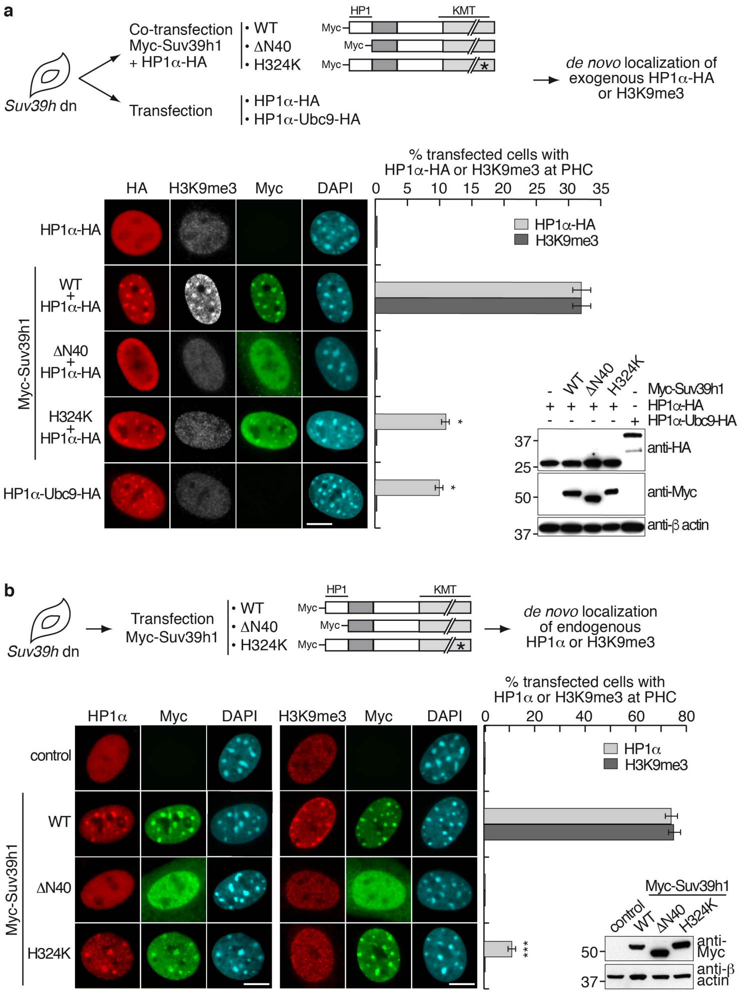

Supplementary Figure 7. H3K9 KMT catalytically dead Suv39h1-H324K promotes *de novo* targeting of HP1α to pericentric heterochromatin.

a. *De novo* localization of exogenous HP1α-HA and H3K9me3 in *Suv39h* double-null cells expressing Myc-Suv39h1-WT, Myc-Suv39h1-ΔN40 or Myc-Suv39h1-H324K analyzed by immunofluorescence using anti-HA (red), anti-H3K9me3 (white) and anti-Myc (green) antibodies 5h after transfection. We examined 300 transfected cells and performed quantitative analysis of the percentage of transfected cells with HP1α-HA or endogenous H3K9me3 enriched at pericentric heterochromatin domains (PHC). Error bars on the graph represent SD from three independent experiments. A comparison of transfected protein expression is shown on the Western blot. *$p<0.05$ (Student's t test). Scale bar, 10 μm. Note that in this assay, we explored whether transfection of *Suv39h* double-null cells with H3K9 KMT catalytically dead Suv39h1-H324K mutant could promote *de novo* localization of exogenous HP1α-HA at pericentric heterochromatin. We used as a positive control Myc-Suv39h1-WT that restores the proper localization of both HP1α and H3K9me3 at pericentric domains [1,2,3], and as a negative control Myc-Suv39h1-ΔN40 that cannot interact with HP1α and does not accumulate at pericentric domains. Transfection with HP1α-HA alone does not lead to its accumulation at pericentric heterochromatin [2]. In 33% of cells co-transfected with Myc-Suv39h1-WT, HP1α-HA localized at pericentric domains and H3K9me3 accumulation was restored. In contrast, we could not detect pericentric localization of HP1α-HA and H3K9me3 in cells transfected with Myc-Suv39h1-ΔN40. Notably, in about 11% of cells co-transfected with H3K9 KMT catalytically dead Myc-Suv39h1-H324K, we found that HP1α-HA accumulated at pericentric domains, although we did not detect H3K9me3 accumulation at these domains. **b**. *De novo* localization of endogenous HP1α and H3K9me3 in *Suv39h* double-null cells expressing Myc-Suv39h1-WT, Myc-Suv39h1-ΔN40 or Myc-Suv39h1-H324K analyzed by immunofluorescence using anti-HP1α (red), anti-H3K9me3 (red) and anti-Myc (green) antibodies 5h after transfection. We performed quantitative analysis of the percentage of transfected cells with endogenous HP1α or H3K9me3 enriched at pericentric heterochromatin domains (PHC). Error bars on the graph represent SD from three independent experiments. A comparison of transfected protein expression is shown on the western blot. ***$p<0.001$ (Student's t test). Scale bar, 10 μm.

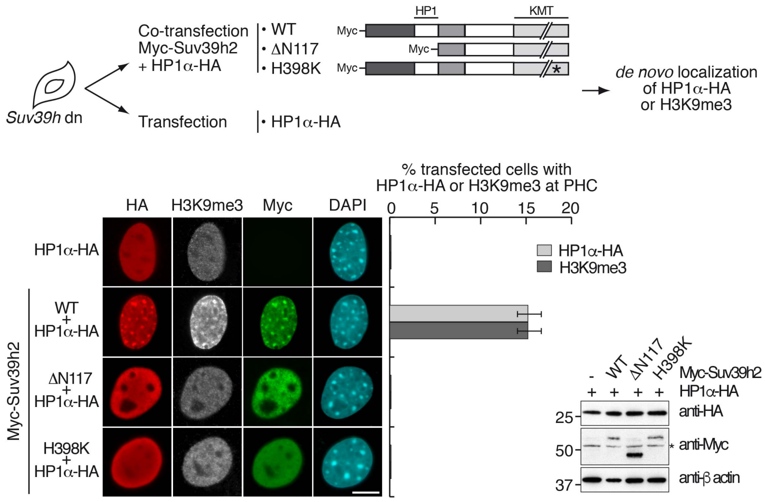

Supplementary Figure 8. Myc-Suv39h2-H398K, a H3K9 KMT catalytically dead mutant of Suv39h2, does not promote *de novo* targeting of HP1α.

De novo localization of HP1α-HA and H3K9me3 in *Suv39h* double-null cells expressing Myc-Suv39h2-WT, Myc-Suv39h2-ΔN117 or Myc-Suv39h2-H398K analyzed by immunofluorescence using anti-HA (red), anti-H3K9me3 (white) and anti-Myc (green) antibodies 5h after transfection. We examined 300 transfected cells and performed quantitative analysis of the percentage of transfected cells with HP1α-HA or endogenous H3K9me3 enriched at pericentric heterochromatin domains (PHC). Error bars on the graph represent SD from three independent experiments. A comparison of transfected protein expression is shown on the western blot. The asterisk indicates unspecific bands. Scale bar, 10 μm.

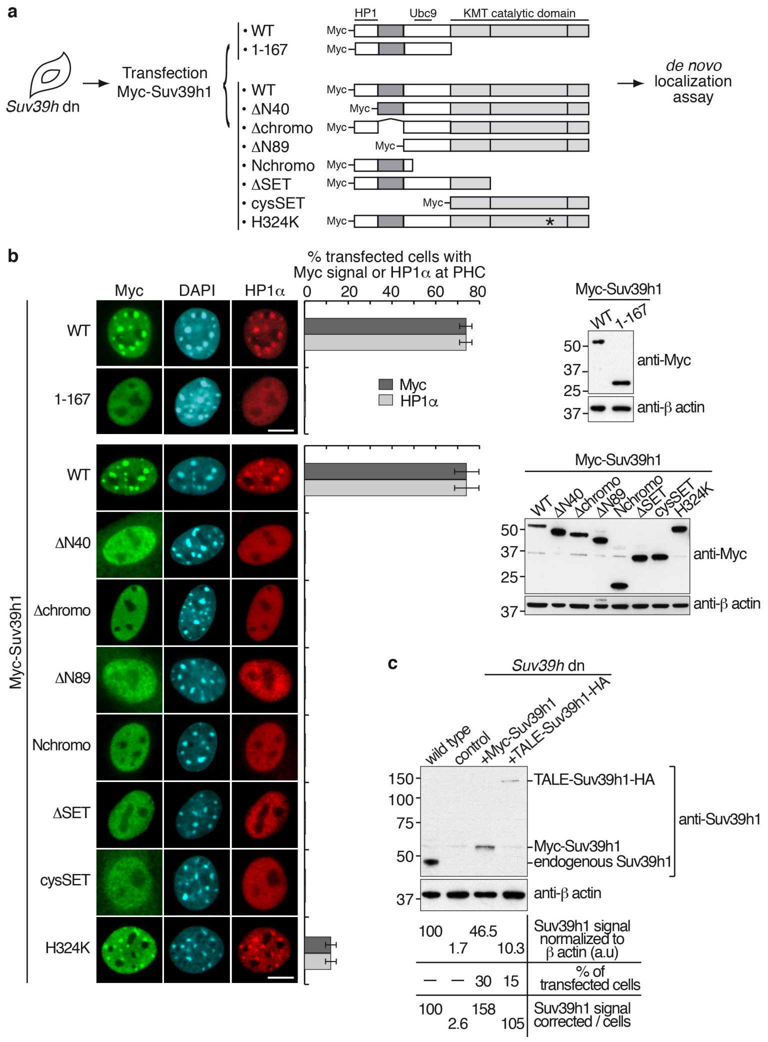

Supplementary Figure 9. ***De novo*** **localization of the Myc-Suv39h1 truncated proteins.**

a. Experimental scheme. **b**. *De novo* localization of Myc-Suv39h1-WT or truncated versions and endogenous HP1α in *Suv39h* double-null cells analyzed by immunofluorescence using anti-Myc (green) and anti- HP1α (red) antibodies 5h after transfection. We performed quantitative analysis of the percentage of transfected cells with the Myc signal or endogenous HP1α enriched at pericentric heterochromatin domains (PHC). Error bars on the graph represent SD from three independent experiments. A comparison of transfected protein expression is shown on the western blots. Scale bar, 10 μm. **c**. Western blot using anti-Suv39h1 antibody to detect Suv39h1 in total extracts prepared from wild type MEFs, *Suv39h* double-null MEFs, *Suv39h* double-null MEFs transfected with Myc-Suv39h1 or TALE-Suv39h1-HA. To estimate how expression of endogenous Suv39h1 in wild type MEFs compared to exogenous Suv39h1 tagged either with Myc or TALE and HA in *Suv39h* double-null MEFs, we adjusted the signal corresponding to exogenous Suv39h1 on the blot to the fraction of transfected cells. In this way, while we cannot exclude variation from cell to cell, we could estimate that on average for transfected cells, exogenous Myc-Suv39h1 expression reaches is 1.5-fold higher than the endogenous normal amount and exogenous TALE-Suv39h1-HA is comparable.

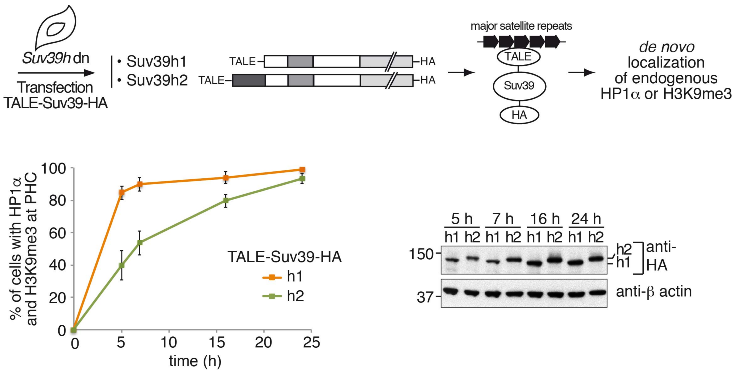

Supplementary Figure 10. *De novo* targeting of HP1α to pericentric heterochromatin in the presence of tethered Suv39h1 or Suv39h2.

Analysis over time of the *de novo* localization of endogenous HP1α following transfection with TALE-Suv39h1-HA or TALE-Suv39h2-HA in *Suv39h* double-null cells. The percentage of cells with HP1α and H3K9me3 enriched at pericentric heterochromatin domains (PHC) as a function of time after transfection is represented. Error bars indicate SD of three independent experiments (300 transfected cells counted in each condition). A comparison of transfected protein expression is shown on the Western blot.

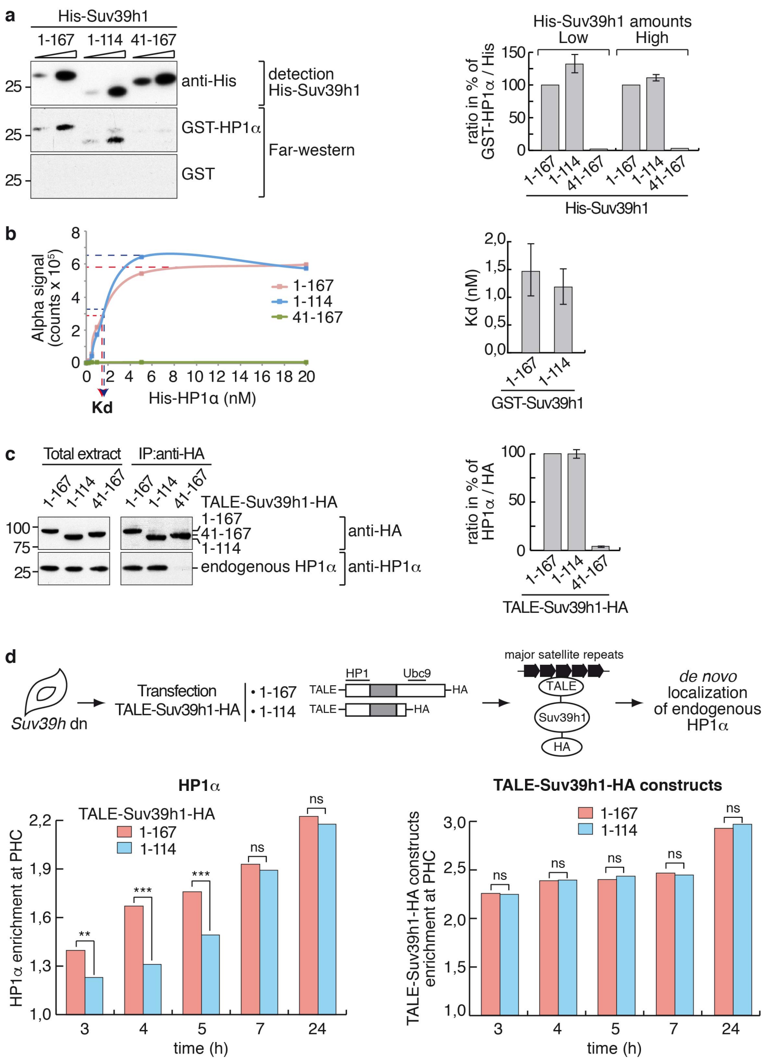

Supplementary Figure 11. Tethering the 1-167 domain of Suv39h1 to pericentric heterochromatin boosts *de novo* targeting of HP1α.

a. Far-western blot using respectively two amounts of His-Suv39h1 truncated versions as baits and purified recombinant GST-HP1α or GST control as preys. We revealed bound GST-HP1α using anti-GST antibodies. The histogram shows the signal of GST-HP1α relative to His signal (ratio) for the two amounts of His-Suv39h1 truncated versions. 100% is set for the His-Suv39h1-1-167. Error bars represent SD from 2 independent experiments. **b**. Saturation binding assay (AlphaSreen) using various concentrations (nM) of His-HP1α and 5 nM of GST-Suv39h1 truncated versions. The affinity constant (Kd) was derived from the saturation curves as the His-HP1α concentration at which half saturating signal is reached (arrows). The histogram shows the mean Kd for the interaction between HP1α and Suv39h1-1-167 (1,47 nM) or Suv-39h1-1-114 (1,18 nM). Error bars represent SD from 3 independent experiments. **c**. Immunoprecipitation of TALE-Suv39h1-1-167-HA, TALE-Suv39h1-1-114-HA and TALE-Suv39h1-41-167-HA. Western blot analysis using anti-HA and anti-HP1α antibodies revealed HA-tagged proteins and endogenous HP1α in total cell extracts and immunoprecipitates. The histogram shows the signal of endogenous HP1α relative to HA signal (ratio). 100% is set for the TALE-Suv39h1-1-167-HA. Error bars represent SD from 3 independent experiments. **d**. Time course analysis of the *de novo* localization of endogenous HP1α in *Suv39h* double-null cells transfected with TALE-Suv39h1-1-167-HA or TALE-Suv39h1-1-114-HA. Top: experimental scheme. Bottom: We performed quantitative analysis of endogenous HP1α (left) and TALE-Suv39h1-HA constructs 1-167 and 1-114 (right) enrichment at pericentric heterochromatin domains (PHC) on at least 100 transfected cells for each condition from four independent experiments using the 3D-FIED method. ns: not significant; $^{**}p<0.01$; $^{***}p<0.001$ (Student's t test).

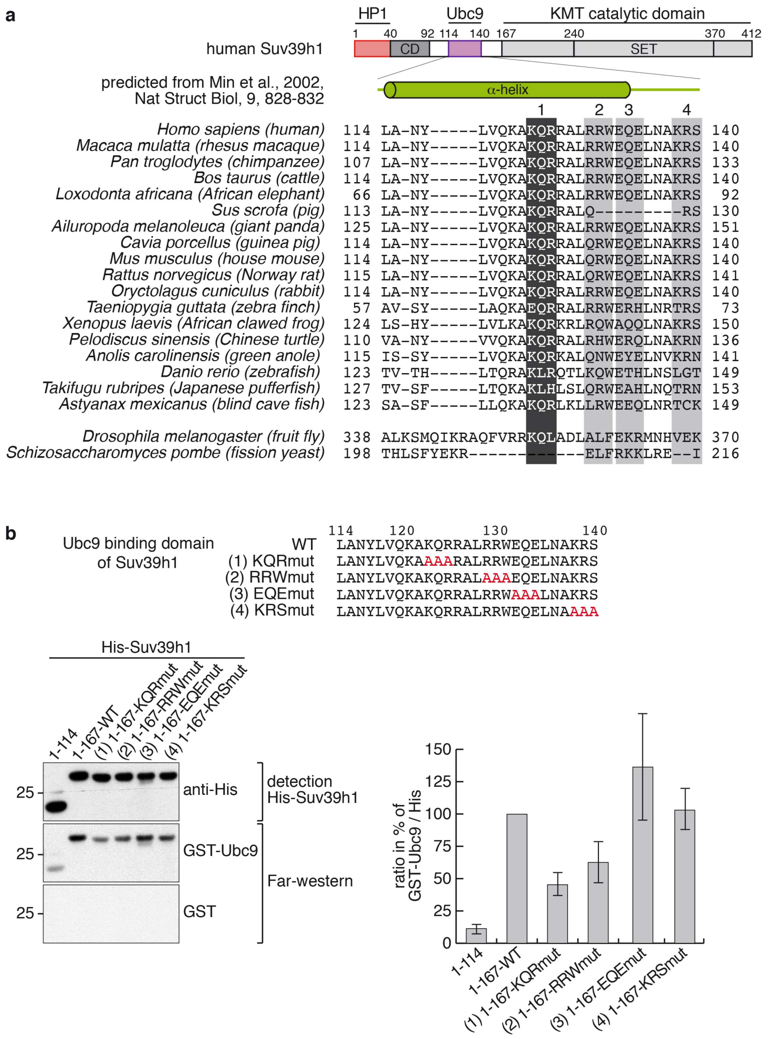

Supplementary Figure 12. The Ubc9 binding domain of Suv39h1.

a. Phylogenetic comparison of Suv39h1 in the Ubc9 binding domain. This domain in human Suv39h1 corresponds to aa114-140 (Supplementary Fig. 4c). It is predicted to fold in a α-helix in line with the structural data of Clr4 [4]. **b**. Far-western blot using His-Suv39h1-1-167-WT or mutated versions (KQRmut, RRWmut, EQEmut or KRSmut) as baits and purified recombinant GST-Ubc9 or GST control as preys. We revealed bound GST-Ubc9 using anti-GST antibodies. The histogram shows the signal of GST-Ubc9 relative to His signal (ratio). 100% is set for the His-Suv39h1-1-167-WT. Error bars represent SD from 2 independent experiments.

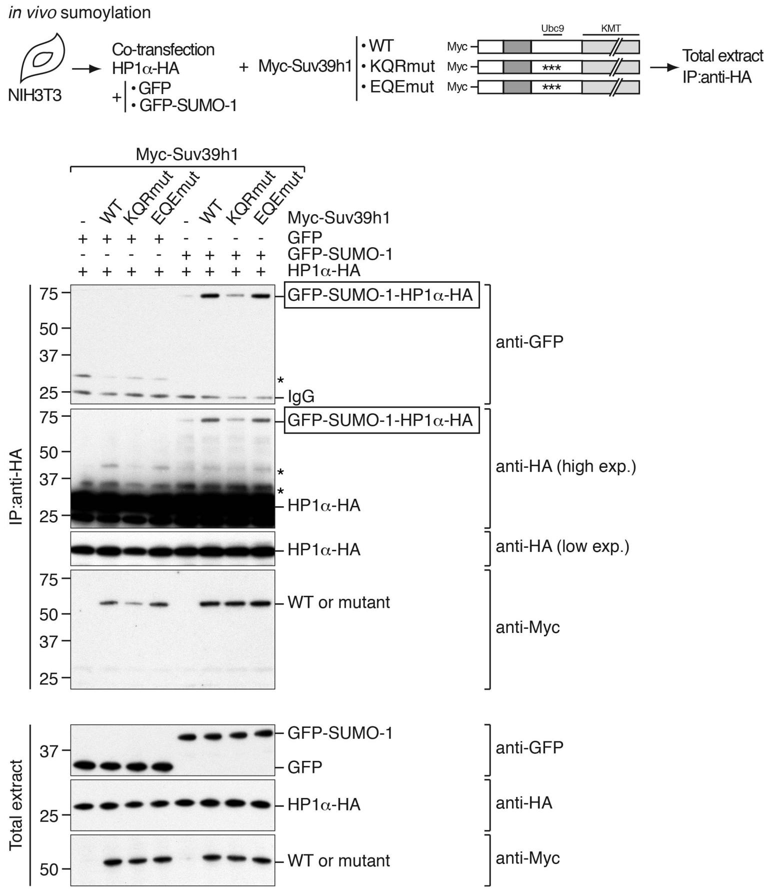

Supplementary Figure 13. Myc-Suv39h1-KQRmut, a mutant unable to bind to Ubc9, does not enhance HP1α sumoylation *in vivo*.

In vivo HP1α sumoylation assay. Top: experimental scheme. Middle: we transfected NIH3T3 cells with HP1α-HA, GFP, GFP-SUMO-1, Myc-Suv39h1-WT, Myc-Suv39h1-KQRmut or Myc-Suv39h1-EQEmut as indicated. We performed anti-HA immunoprecipitation from total extracts. Western blot analysis using anti-GFP and anti-HA antibodies revealed sumoylated HP1α-HA (GFP-SUMO-1-HP1α-HA, box) and unmodified HP1α-HA. Western blot using anti-Myc antibodies indicated that the three Myc-tagged proteins coimmunoprecipitated with HP1α-HA. IgG corresponds to the immunoglobulin light chain. Asterisks indicate unspecific bands. Bottom: We verified the expression levels of all transfected proteins in total cell extracts by Western blot using anti-GFP, anti-HA and anti-Myc antibodies.

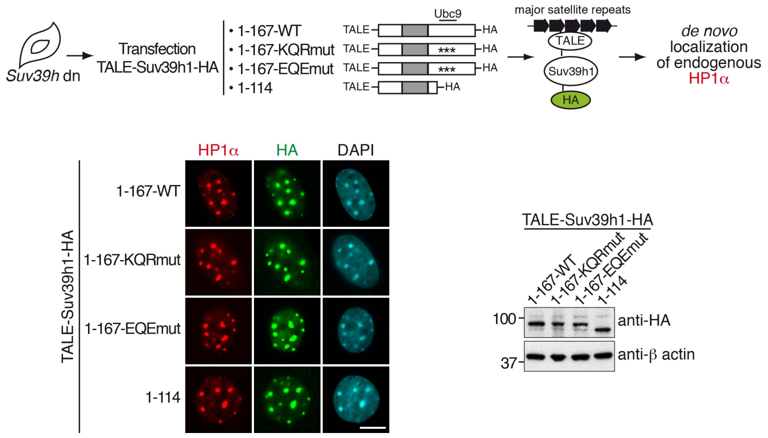

Supplementary Figure 14. Tethering Suv39h1-1-167-KQR mutant to pericentric heterochromatin does not promote an efficient *de novo* targeting of HP1α.

Top: experimental scheme. Bottom: *De novo* localization of endogenous HP1α (red) in *Suv39h* double-null cells expressing TALE-Suv39h1-1-167-WT, TALE-Suv39h1-1-167-KQRmut, TALE-Suv39h1-1-167-EQEmut or TALE-Suv39h1-1-114 tagged with HA (green) analyzed by immunofluorescence 5h after transfection. We observed that the transfection of TALE-Suv39h1-1-167-KQRmut gives rise to a high mortality of cells compared to the others transfected proteins. A comparison of transfected protein expression is shown on the Western blot. Scale bar, 10 μm.

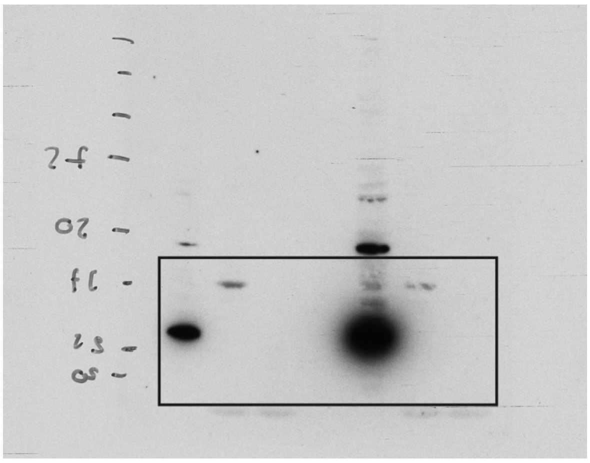

Supplementary Figure 15: Uncropped Western blot corresponding to figure 1b.
Box indicates portion of the blot displayed in figure 1b.

.

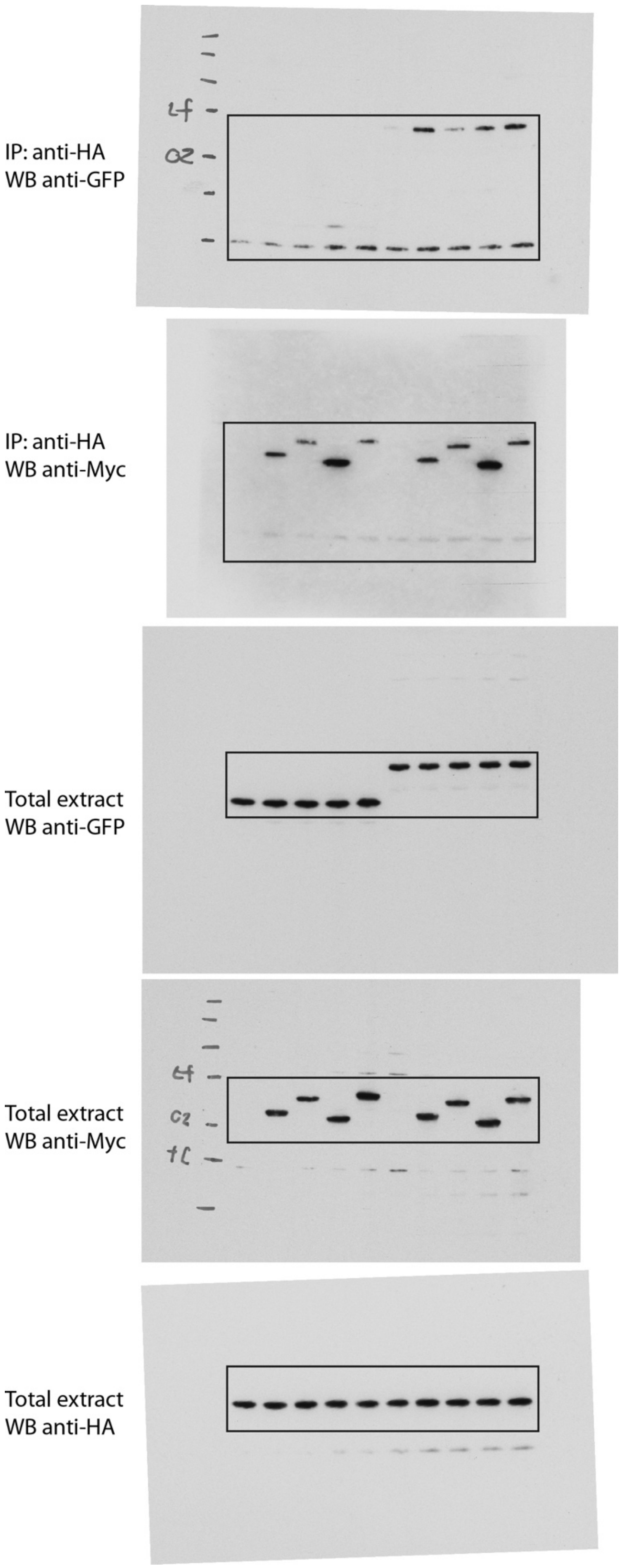

Supplementary Figure 16: Uncropped Western blots corresponding to figure 1d.
Boxes indicate portions of the blots displayed in figure 1d.

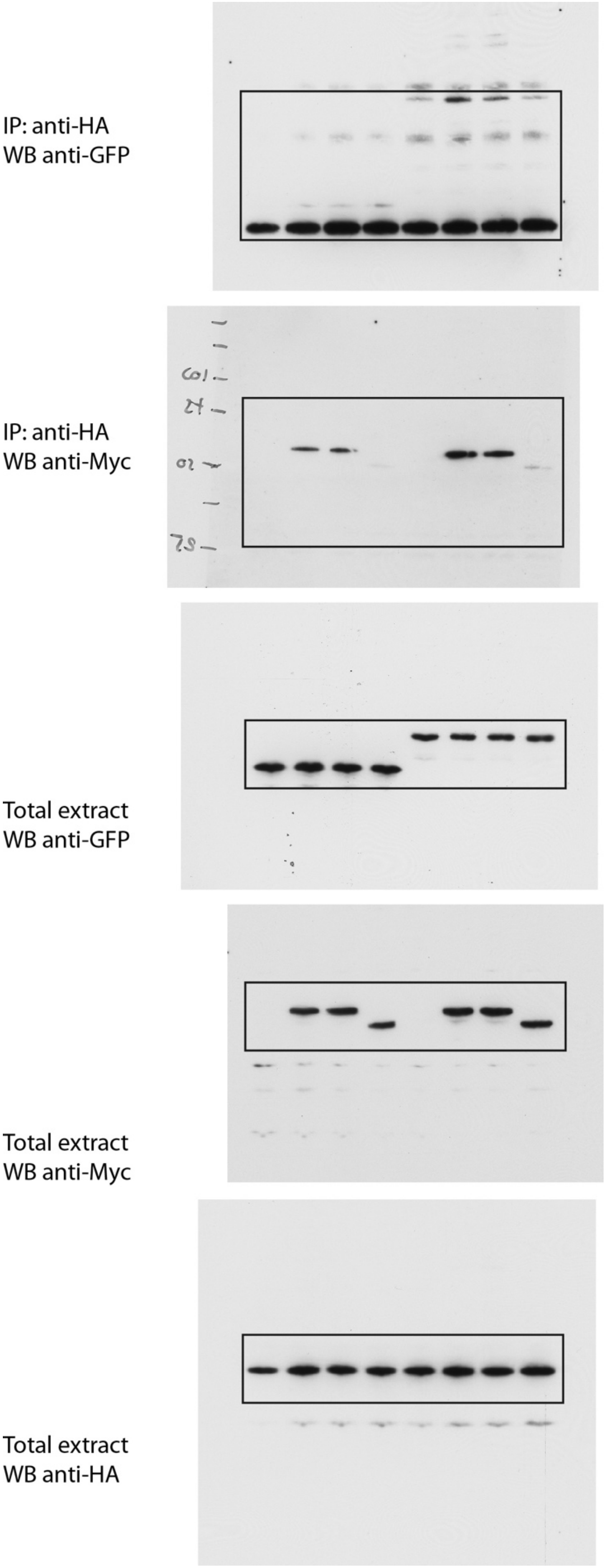

Supplementary Figure 17: Uncropped Western blots corresponding to figure 1e.
Boxes indicate portions of the blots displayed in figure 1e.

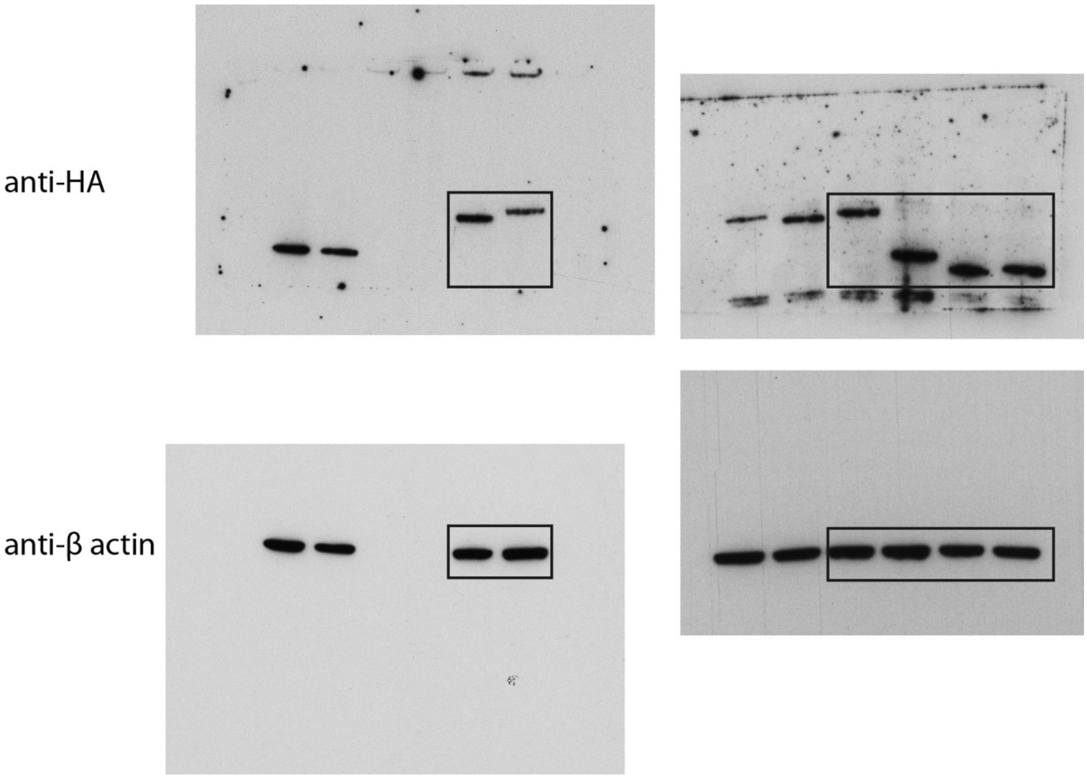

Supplementary Figure 18: Uncropped Western blots corresponding to figure 3b. Boxes indicate portions of the blots displayed in figure 3b.

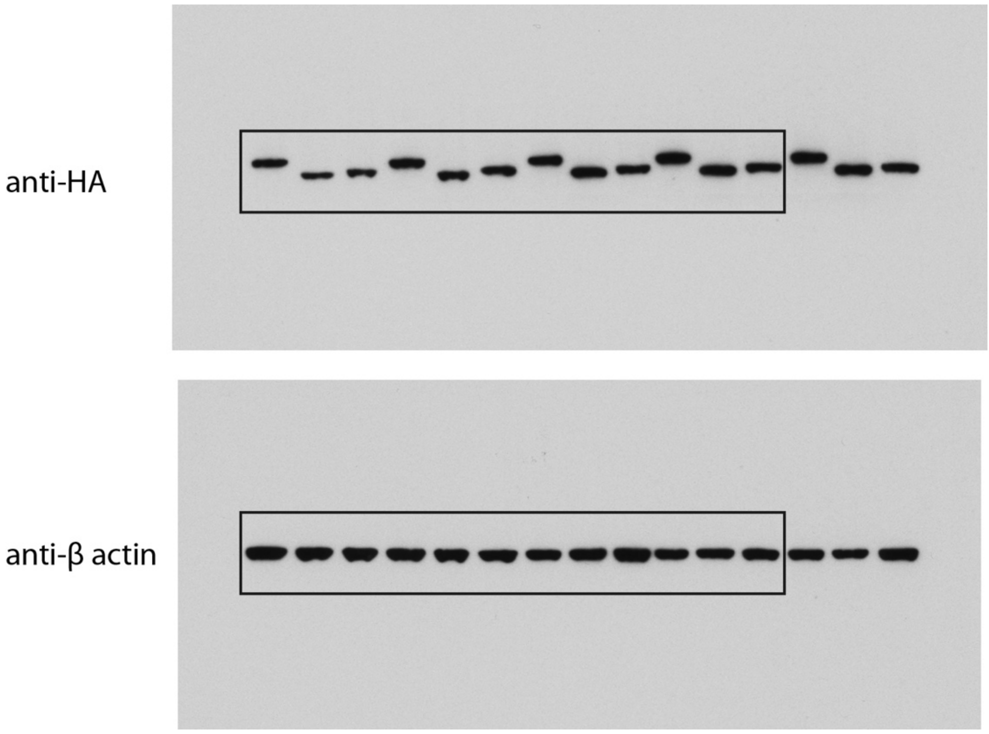

Supplementary Figure 19: Uncropped Western blots corresponding to figure 3c.
Boxes indicate portions of the blots displayed in figure 3c.

Supplementary references

1 Lachner, M., O'Carroll, D., Rea, S., Mechtler, K. & Jenuwein, T. Methylation of histone H3 lysine 9 creates a binding site for HP1 proteins. *Nature* **410**, 116-120 (2001).
2 Maison, C. *et al.* SUMOylation promotes de novo targeting of HP1alpha to pericentric heterochromatin. *Nat Genet* **43**, 220-227 (2011).
3 Loyola, A., Bonaldi, T., Roche, D., Imhof, A. & Almouzni, G. PTMs on H3 variants before chromatin assembly potentiate their final epigenetic state. *Mol Cell* **24**, 309-316 (2006).
4 Min, J., Zhang, X., Cheng, X., Grewal, S. I. & Xu, R. M. Structure of the SET domain histone lysine methyltransferase Clr4. *Nat Struct Biol* **9**, 828-832 (2002).
