# Supplementary material for: The methyltransferase Suv39h1 links the SUMO pathway to HP1α marking at pericentric heterochromatin
Source: Nat Commun. 2016 Jul 18;7:12224. doi: 10.1038/ncomms12224 (PMC4960310; doi:10.1038/ncomms12224)
Supplement: Peer Review File [file ncomms12224-s2.pdf]

Editorial Note: this manuscript has been previously reviewed at another journal that is not operating a transparent peer review scheme. This document only contains reviewer comments and rebuttal letters for versions considered at Nature Communications.
